# Prognostic significance of infarct core pathology revealed by quantitative non-contrast in comparison with contrast cardiac magnetic resonance imaging in reperfused ST-elevation myocardial infarction survivors

**DOI:** 10.1093/eurheartj/ehv372

**Published:** 2015-08-10

**Authors:** David Carrick, Caroline Haig, Sam Rauhalammi, Nadeem Ahmed, Ify Mordi, Margaret McEntegart, Mark C. Petrie, Hany Eteiba, Stuart Hood, Stuart Watkins, Mitchell Lindsay, Ahmed Mahrous, Ian Ford, Niko Tzemos, Naveed Sattar, Paul Welsh, Aleksandra Radjenovic, Keith G. Oldroyd, Colin Berry

**Affiliations:** 1 BHF Glasgow Cardiovascular Research Centre, Institute of Cardiovascular and Medical Sciences, University of Glasgow, Glasgow G128TA, UK; 2 West of Scotland Heart and Lung Centre, Golden Jubilee National Hospital, Clydebank, UK; 3 Robertson Centre for Biostatistics, University of Glasgow, Glasgow, UK

**Keywords:** ST-elevation myocardial infarction, Percutaneous coronary intervention, Cardiac magnetic resonance, Adverse remodelling

## Abstract

**Aims:**

To assess the prognostic significance of infarct core tissue characteristics using cardiac magnetic resonance (CMR) imaging in survivors of acute ST-elevation myocardial infarction (STEMI).

**Methods and results:**

We performed an observational prospective single centre cohort study in 300 reperfused STEMI patients (mean ± SD age 59 ± 12 years, 74% male) who underwent CMR 2 days and 6 months post-myocardial infarction (*n* = 267). Native T1 was measured in myocardial regions of interest (*n* = 288). Adverse remodelling was defined as an increase in left ventricular (LV) end-diastolic volume ≥20% at 6 months. All-cause death or first heart failure hospitalization was a pre-specified outcome that was assessed during follow-up (median duration 845 days). One hundred and sixty (56%) patients had a hypo-intense infarct core disclosed by native T1. In multivariable regression, infarct core native T1 was inversely associated with adverse remodelling [odds ratio (95% confidence interval (CI)] per 10 ms reduction in native T1: 0.91 (0.82, 0.00); *P* = 0.061). Thirty (10.4%) of 288 patients died or experienced a heart failure event and 13 of these events occurred post-discharge. Native T1 values (ms) within the hypo-intense infarct core (*n* = 160 STEMI patients) were inversely associated with the risk of all-cause death or first hospitalization for heart failure post-discharge (for a 10 ms increase in native T1: hazard ratio 0.730, 95% CI 0.617, 0.863; *P* < 0.001) including after adjustment for left ventricular ejection fraction, infarct core T2 and myocardial haemorrhage. The prognostic results for microvascular obstruction were similar.

**Conclusion:**

Infarct core native T1 represents a novel non-contrast CMR biomarker with potential for infarct characterization and prognostication in STEMI survivors. Confirmatory studies are warranted.

**ClinicalTrials.gov identifier:**

NCT02072850.

**See page 1060 for the editorial comment on this article (doi:10.1093/eurheartj/ehv517)**

**Clinical perspective**

Contrast-enhanced cardiac magnetic resonance (CMR) imaging is the established approach for imaging infarct pathology in survivors of ST-elevation myocardial infarction (STEMI). The pathophysiological and prognostic importance of infarct pathology disclosed by non-contrast native T1 CMR mapping in acute STEMI patients is unknown. We performed a prospective single centre cohort study in 300 reperfused STEMI patients who underwent CMR 2 days and 6 months (*n* = 267) post-myocardial infarction and clinical follow-up (median duration 2.5 years). Infarct core pathology revealed by native T1 mapping was feasible [*n* = 288 (96%) with evaluable data] and had superior prognostic value compared with infarct core T2 and myocardial haemorrhage, and similar prognostic value compared with microvascular obstruction revealed by late gadolinium enhancement CMR. Infarct core native T1 is a novel non-contrast CMR biomarker with potential for infarct characterization and prognostication in STEMI survivors.

## Introduction

Myocardial infarct size^1,2^ and microvascular obstruction^3–5^ revealed by contrast-enhanced cardiac magnetic resonance (CMR) reflect the efficacy of reperfusion therapy and are prognostically important findings in survivors of ST-elevation myocardial infarction (STEMI).

Human tissue has fundamental magnetic properties, including the longitudinal (spin-lattice) relaxation time (native T1 in milliseconds). Native T1 is influenced by water content, binding with macromolecules (water mobility), and cell content.^6,7^ Native T1 CMR does not involve an intravenous contrast agent. Tissue water content increases as a result of ischaemia and longer T1 times may represent a biomarker of localized myocardial injury.^8–16^

The clinical significance of tissue changes within the infarct core in patients with acute reperfused STEMI has not been directly assessed. We hypothesized that baseline native T1 values would be (i) inversely associated with the severity of MI, including microvascular obstruction, (ii) independently associated with left ventricular (LV) remodelling, and (iii) independently associated with pre-defined health outcomes. Should these hypotheses be confirmed then infarct core native T1 mapping without an intravenous contrast agent might have potential as an alternative biomarker to microvascular obstruction revealed by contrast-enhanced CMR.

To investigate these hypotheses, we measured native T1 in myocardial regions of interest in STEMI patients undergoing serial CMR imaging 2 days and 6 months post-MI. We assessed the clinical associates of native T1 within the hypo-intense infarct core and subsequent LV remodelling and examined its association with all-cause death and first hospitalization for heart failure.

## Methods

### Study population and ST-elevation myocardial infarction management

We performed an observational prospective CMR cohort study in a single regional cardiac centre between 14 July 2011 and 22 November 2012. Three hundred and forty three STEMI patients provided written informed consent to undergo CMR 2 days and 6 months post-MI. Patients were eligible if they had an indication for primary percutaneous coronary intervention (PCI) or thrombolysis for acute STEMI due to a history of symptoms consistent with acute myocardial ischaemia and with supporting changes on the electrocardiogram (ECG) (i.e. ST-segment elevation or new left bundle-branch block).^17^ Exclusion criteria represented standard contra-indications to contrast CMR, including a pacemaker and estimated glomerular filtration rate <30 mL/min/1.73 m^2^. The study was approved by the National Research Ethics Service and all participants provided written informed consent. Acute STEMI management followed contemporary guidelines.^17,18^ Aspiration thrombectomy, direct stenting, anti-thrombotic drugs, and other therapies were administered according to clinical judgment (Supplementary material online, Methods). The ClinicalTrials.gov identifier is NCT02072850.

### Cardiac magnetic resonance acquisition

Cardiac magnetic resonance was performed on a Siemens MAGNETOM Avanto (Erlangen, Germany) 1.5-Tesla scanner with a 12-element phased array cardiac surface coil.^19^ The imaging protocol included cine magnetic resonance imaging with steady-state free precession (SSFP), native T1 mapping,^15,20^ T2 mapping,^21,22^ T2*-mapping, and delayed-enhancement phase-sensitive inversion-recovery pulse sequences.^23^ The scan acquisitions were spatially co-registered and also included different slice orientations to enhance diagnostic confidence. Cardiac magnetic resonance was also performed in 50 healthy volunteers of similar age and gender in order to obtain local reference values for myocardial native T1 (Supplementary material online). Patients and healthy volunteers underwent the same imaging protocol except that healthy volunteers <45 years did not receive gadolinium. The coefficients of variation for native T1 were also measured (Supplementary material online, Results).

Native T1 maps were acquired in three short-axial slices (basal, mid, and apical), using an optimized modified look-locker inversion recovery (MOLLI) T1-mapping investigational prototype sequence^15,20^ before contrast administration (Supplementary material online, Methods; work-in-progress 448, Siemens Healthcare). The MOLLI T1 cardiac-gated acquisition involved three inversion recovery prepared Look-Locker experiments combined within one protocol.^15^ The CMR parameters were: bandwidth ∼1090 Hz/pixel, flip angle 35°, echo time (TE) 1.1 ms, T1 of first experiment 100 ms, TI increment 80 ms, matrix 192 × 124 pixels, spatial resolution 2.2 × 1.8 × 8.0 mm, slice thickness 8 mm, and scan time 17 heartbeats. The prototype pulse sequence did not involve motion correction.

T2 maps were acquired in contiguous short-axis slices covering the whole ventricle, using an investigational prototype T2-prepared TrueFisp sequence^21,22^ (Supplementary material online, Methods). Typical imaging parameters were: bandwidth ∼947 Hz/pixel, flip angle 70°, T2 preparations: 0, 24, and 55 ms, respectively, matrix 160 × 105 pixels, spatial resolution 2.6 × 2.1 × 8.0 mm, and slice thickness 8 mm.

T2*-maps were obtained using an investigational prototype T2* map sequence acquired in three short-axis slices (basal, mid, and apical). Typical imaging parameters were: bandwidth ∼814 (8×) Hz/pixel; flip angle 18°; matrix 256 × 115; spatial resolution 2.6 × 1.6 × 10 mm; slice thickness 8 mm.

Early gadolinium enhancement (EGE) imaging was acquired 1, 3, 5, and 7 min post-contrast injection using a TrueFISP readout and fixed inversion time (TI) of 440 ms. Late gadolinium enhancement images covering the entire LV were acquired 10–15 min after IV injection of 0.15 mmol/kg of gadoterate meglumine (Gd^2+^-DOTA, Dotarem, Guebert S.A.) using segmented phase-sensitive inversion recovery turbo fast low-angle shot sequence.^23^ Typical imaging parameters were: matrix = 192 × 256, flip angle = 25°, TE = 3.36 ms, bandwidth = 130 Hz/pixel, echo spacing = 8.7 ms, and trigger pulse = 2. The voxel size was 1.8 × 1.3 × 8 mm^3^. Inversion times were individually adjusted to optimize nulling of apparently normal myocardium (typical values, 200–300 ms).

### Cardiac magnetic resonance analyses

The images were analysed on a Siemens work-station by observers with at least 3 years CMR experience (N.A., D.C., I.M, and S.R.). All of the images were reviewed by experienced CMR cardiologists (C.B. and N.T.). Left ventricular dimensions, volumes, and ejection fraction were quantified using computer-assisted planimetry (syngo MR^®^, Siemens Healthcare, Erlangen, Germany). The late gadolinium enhancement images were analysed for infarct size and microvascular obstruction by observers (N.A. and I.M.) who were blinded to all of the other data. In healthy volunteers, the absence of late gadolinium enhancement was determined qualitatively by visual assessment.

#### Native T1 mapping: standardized measurements in myocardial regions of interest

**Figure 1:**
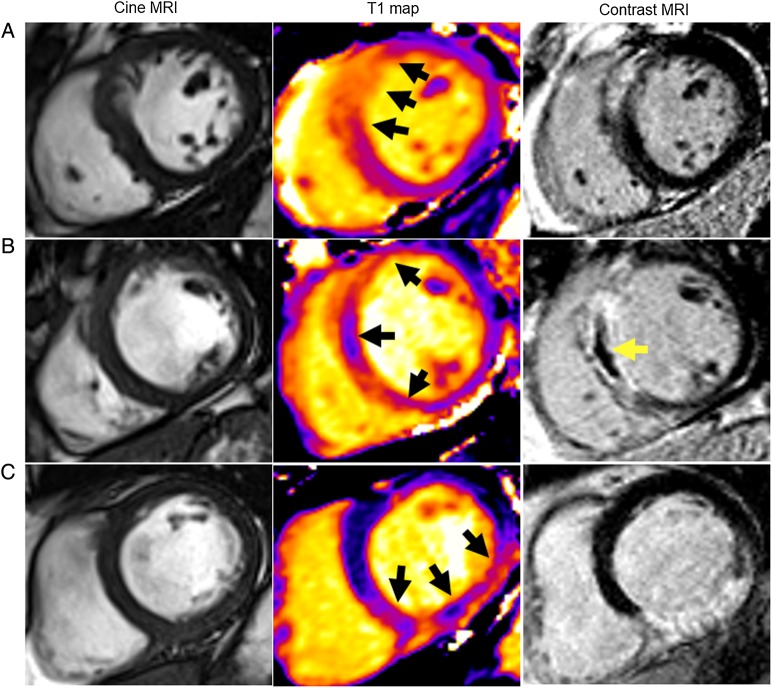
Three patients with acute ST-elevation myocardial infarction treated by primary PCI and with the same anti-thrombotic therapies, including aspirin, clopidogrel, heparin, and intravenous tirofiban. Each patient had normal thrombolysis in myocardial infarction Grade 3 flow at the end of PCI. Cardiac magnetic resonance imaging was performed for each patient 2 days later. (*A*) Patient with no T1 hypo-intense infarct core and no microvascular obstruction*.* Native T1 within the injury zone (*middle*) measured 1211 ms. Acute infarct size revealed by late gadolinium enhancement (*right*) was 22.2%. The left ventricular ejection fraction and left ventricular end-diastolic volume were 55.2% and 143.1 mL, respectively. Analysis of the repeat magnetic resonance imaging scan after 6 months follow-up indicated that the final infarct size was 15.6% of left ventricular mass and the left ventricular end-diastolic volume had reduced to 103.0 mL. This patient had an uncomplicated clinical course. (*B*) Patient with both T1 hypo-intense infarct core and microvascular obstruction*.* T1 mapping (*middle*) revealed a hypo-intense region within the infarct core, corresponding to the area of microvascular obstruction on contrast-enhanced magnetic resonance imaging (*right*). Native T1 within the infarct core measured 1036 ms, which was substantially lower than the T1 value measured at the periphery of the infarct zone (1193 ms). Acute infarct size revealed by late gadolinium enhancement (*right*) was 33.0%. Microvascular obstruction depicted as the central dark zone within the infarct territory was 3.6% of left ventricular mass. The left ventricular ejection fraction and end-diastolic volume were 45.8% and 199.3 mL, respectively. The final infarct size at 6 months was 22.6% of left ventricular mass and the left ventricular end-diastolic volume had increased to 221.8 mL. This patient was re-hospitalized for new onset heart failure during follow-up.

Native T1 mapping is a CMR method providing a parametric colour-encoded anatomical map in which the T1 value is encoded in each pixel^24^ (*Figure 1*). The native T1 map analyses were informed by contemporary CMR guidelines.^24^ Left ventricular contours were delineated with computer-assisted planimetry on the raw T1 image and copied onto the colour-encoded spatially co-registered map. Apical segments were not included because of partial volume effects. Particular care was taken to delineate regions of interest with adequate margins of separation from tissue interfaces prone to partial volume averaging such as between myocardium and blood.^19,24,25^ Each T1 map image was assessed for the presence of artefacts relating to susceptibility effects, or cardio-respiratory motion. Each colour map was evaluated against the original images. When artefacts occurred the affected segments were not included in the analysis.

In STEMI patients, myocardial T1 values were segmented spatially and regions of interest were defined as (i) remote myocardium, (ii) injured myocardium, and (iii) infarct core. The regions of interest were planimetered to include the entire area of interest with distinct margins of separation from tissue interfaces to avoid partial volume averaging. The remote myocardium region of interest was defined as myocardium 180° from the affected zone with no visible evidence of infarction, oedema, or wall motion abnormalities (assessed by inspecting corresponding contrast-enhanced T1-weighted, T2-weighted, and cine images, respectively). The infarct zone region of interest was defined as myocardium with pixel values (T1 or T2) >2 SD from remote myocardium on T2-weighted CMR.^21,22^ The hypo-intense infarct core was defined as an area in the centre of the infarct territory having a mean T1 value of at least 2 standard deviations (SDs) below the T1 value of the periphery of the area at risk.^21,22^ The assessment of T1 maps and adjudication (present/absent) of a hypo-intense core was performed independently by D.C.

In healthy volunteers, the mid-ventricular T1 colour-encoded map was segmented into six equal segments, using the anterior right ventricular-LV insertion point as the reference point.^26^ T1 was measured in each of these segments, and regions of interest were planimetered distinct and separate from blood-pool and tissue interfaces. These segmental values were also averaged to provide one value per subject. Results are presented as average values for segments and slices.

#### Infarct definition and size

The presence of acute infarction was established based on abnormalities in cine wall motion, rest first-pass myocardial perfusion, and delayed-enhancement imaging in two imaging planes. In addition, supporting changes on the ECG and coronary angiogram were also required. Acute infarction was considered present only if late gadolinium enhancement was confirmed on both the axial and long-axis acquisitions. The myocardial mass of late gadolinium (grams) was quantified using computer-assisted planimetry and the territory of infarction was delineated using a signal-intensity threshold of >5 SDs above a remote reference region and expressed as a percentage of total LV mass.^27^ Infarct regions with evidence of microvascular obstruction were included within the infarct area and the extent of microvascular LV ventricular mass was also measured. The measurements of infarct size were performed by I.M. and N.A.

#### Microvascular obstruction

Microvascular obstruction was defined as a dark zone on EGE imaging 1, 3, 5, and 7 min post-contrast injection that remained present within an area of LGE at 15 min. Identification of microvascular obstruction was performed independently by I.M. and N.A.

#### Area-at-risk

Area-at-risk was defined as LV myocardium with pixel values (T2) >2 SDs from remote myocardium.^4,21,22,28–30^ In order to assess the area-at-risk, the epi- and endocardial contours on the last corresponding T2-weighted raw image with an echo time of 55 ms were planimetered.^21^ Contours were then copied to the computed T2 map and corrected when necessary by consulting the SSFP cine images.

#### Myocardial salvage

Myocardial salvage was calculated by subtraction of per cent infarct size from per cent area at risk.^4,30,31^ The myocardial salvage index was calculated by dividing the myocardial salvage area by the initial area at risk.

#### Adverse remodelling

Adverse remodelling was pre-defined as an increase in LV end-diastolic volume ≥20% at 6 months from baseline.^3^

#### Myocardial haemorrhage

On the T2* maps, a region of reduced signal intensity within the infarcted area, with a T2* value of <20 ms^32–35^ was considered to confirm the presence of myocardial haemorrhage.

### Electrocardiogram

A 12-lead ECG was obtained before coronary reperfusion and 60 min afterwards. The extent of ST-segment resolution on the ECG assessed 60 min after reperfusion compared with the baseline ECG before reperfusion^17^ was expressed as complete (≥70%), incomplete (30% to <70%), or none (≤30%).

### Laboratory analyses

The acquisition of the ECGs and blood samples for biochemical and hematologic analyses are described in Supplementary material online, Methods.

### Pre-specified health outcomes

We pre-specified adverse health outcomes that are pathophysiologically linked with STEMI. The primary composite outcome was (i) all-cause death or first heart failure hospitalization (Supplementary material online, Methods). Other health outcomes included major adverse cardiac events (MACEs) defined as cardiac death, non-fatal MI, or hospitalization for heart failure.

**Figure 2:**
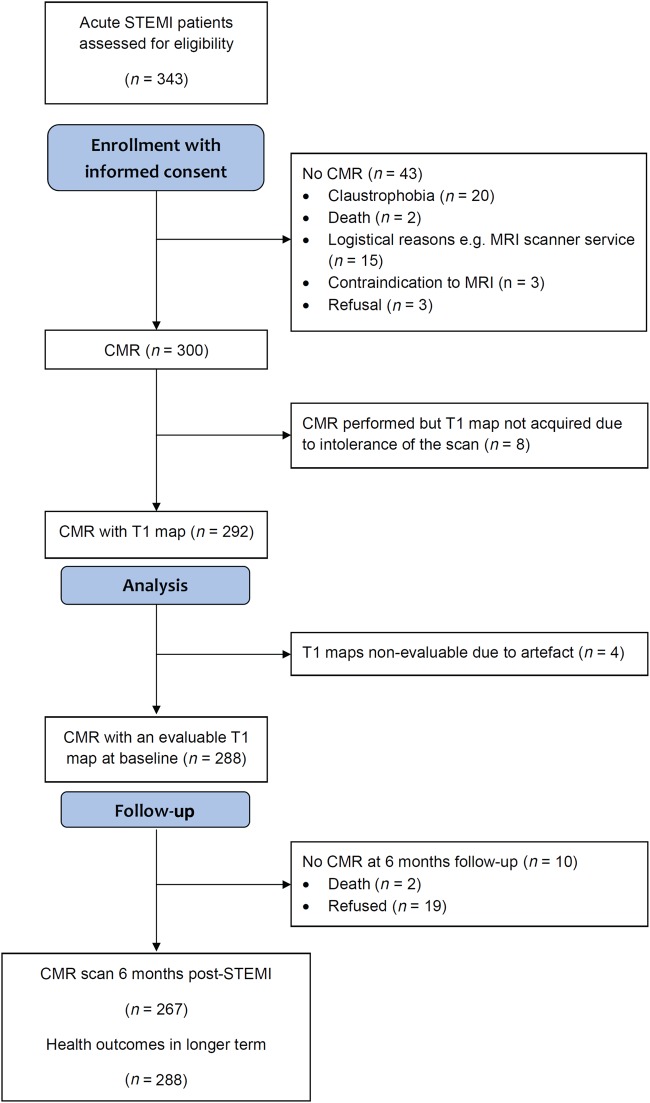
Flow diagram of the cohort study.

Research staff screened for events from enrolment by checking the medical records and by contacting patients and their primary and secondary care physicians, as appropriate with no loss to follow-up (*Figure 2*). Each serious adverse event (SAE) was reviewed by a cardiologist who was independent of the research team and blinded to all of the clinical and CMR data. The SAEs were defined according to standard guidelines^36,37^ (Supplementary material online, Methods) and categorized as having occurred either during the index admission or post-discharge. All study participants were followed up for a minimum of 18 months after discharge. The median duration of follow-up was of 845 days [post-discharge censor duration (range) 598–1098 days].

### Statistical analyses

The sample size calculation is described in Supplementary material online, Methods. We estimated that at least 30 MACE events would occur based on a conservative estimate of the event rate (10–12%) at 18 months.

Categorical variables are expressed as number and percentage of patients. Most continuous variables followed a normal distribution and are therefore presented as means together with SD. Those variables that did not follow a normal distribution are presented as medians with interquartile range. Differences in continuous variables between groups were assessed by the Student's *t*-test or analysis of variance (ANOVA) for continuous data with normal distribution, otherwise the non-parametric Wilcoxon rank sum test or Kruskal–Wallis test. Differences in categorical variables between groups were assessed using a χ^2^ test or Fisher's test, as appropriate. Correlation analyses were Pearson or Spearman tests, as indicated. Random effects models were used to compute inter- and intra-rater reliability measures [intra-class correlation coefficient (ICC)] for the reliability of infarct core native T1 values measured independently by 2 observers in 12 randomly selected patients from the cohort.

Univariable and multivariable linear regression methods to identify associates of T1 values for (i) remote myocardium, (ii) injured myocardium within the area at risk, (iii) infarct core in all patients, and (iv) in patients without late microvascular obstruction are described in Supplementary material online, Methods.

Receiver-operating curve (ROC), Kaplan–Meier, and Cox proportional hazards methods were used to identify potential clinical predictors of all-cause death/heart failure events and MACE, including patient characteristics, CMR findings, and native T1. The net reclassification improvement (NRI) was calculated as described by Pencina *et al.*^38^

All *P*-values are two sided. *P*-value of<0.05 should be interpreted exploratively. Statistical analyses were performed using *R* version 2.15.1 or SAS v 9.3, or higher versions of these programs.

## Results

Of 343 STEMI patients referred for emergency reperfusion therapy, 300 underwent serial CMR at 1.5 T 2.2 ± 1.9 days and 6 months after hospital admission (*Figure 2*). Two hundred and ninety-two STEMI patients had a T1-map acquisition and 288 (99%) had evaluable T1 data (*Figure 2*). Cardiac magnetic resonance follow-up at 6 months was achieved in 267 (93%) of the patients and the reasons for non-attendance are summarized in *Figure 2*. Information on vital status and SAEs were available in all (100%) of the 288 participants.

### Patient characteristics

*Table 1* shows the characteristics of the patients, including the patients with a hypo-intense infarct core revealed by native T1 mapping [*n* = 160 (56%), grouped by thirds of native T1]. The C-reactive protein and leucocyte results are described in Supplementary material online, Table S1. The characteristics of those patients with missing CMR data at 6 months are described in Supplementary material online, Table S2.

### Left ventricular function and pathology

#### Initial cardiac magnetic resonance findings following hospital admission

The CMR findings are summarized in *Table 2* and case examples are shown in *Figure 2*. At baseline, the mean (SD) myocardial infarct size was 18 (14) % of LV mass. The average infarct core native T1 (997 (57)) was higher than native T1 in the remote myocardium 961 (25) ms; *P* < 0.01] but lower than native T1 in the area at risk (1097 (52) ms; *P* < 0.01). The ICC for T1 core is described in Supplementary material online, Results.

### Baseline associates of infarct core native T1 (Hypothesis 1)

Native T1 in the infarct core was inversely associated with thrombolysis in myocardial infarction (TIMI) coronary flow grades at the end of emergency PCI, Killip class and neutrophil count at initial presentation (all *P* < 0.04), independent of left ventricular ejection fraction (LVEF), LV end-diastolic volume, or infarct size (*Table 3*).

**Table 3:** Associates of infarct core native T1 time (for a 10 ms difference) in 160 ST-elevation myocardial infarction survivors with infarct core pathology revealed by native T1 mapping with cardiac magnetic resonance 2 days post-myocardial infarction

Multiple stepwise regression (for a 10 ms difference in infarct core T1)	Coefficient (95% CI)	*P*-value
A. Including patient characteristics and angiographic data*		
Systolic blood pressure at initial angiography (mmHg)	−0.05 (−0.09, −0.01)	0.007
Killip Class 3 or 4	−3.84 (−6.87, −0.80)	0.014
TIMI flow Grade 2 or 3 post-PCI	−7.51 (−15.42, 0.40)	0.063
B. Including patient characteristics, angiographic data, and minimum neutrophil count*		
Systolic blood pressure at initial angiography (mmHg)	−0.05 (−0.09, −0.01)	0.015
Killip Class 3 or 4	−3.39 (−6.45, −0.33)	0.030
TIMI flow Grade 2 or 3 at the end of PCI	−9.77 (−17.67, −1.87)	0.005
Minimum neutrophil count (×10^9^ L)	−0.50 (−0.86, −0.15)	0.005
C. Including patient characteristics, angiographic data, minimum neutrophil count, and T2 core (1 ms)^a^		
T2 core (1 ms)	0.50 (0.32, 0.67)	<0.001
Neutrophils	−0.39 (−0.71, 0.07)	0.016
Gender (male)	−2.32 (−4.25, 0.39)	0.019
SBP	−0.03 (−0.07, 0.00)	0.059
TIMI 2/3 post-PCI	−5.46 (−12.62, 1.70)	0.134

The coefficient (95% CIs) indicates the magnitude and direction of the effect of the patient characteristic (binary or continuous) on the infarct core T1 (ms). For example, in models A and B, on average, infarct core native T1 (10 ms difference) is 0.50 lower for each 1 mmHg increase in SBP.

^a^The clinical and angiographic characteristics that were assessed are listed in *Table 1*. The univariable associates with native T1 in the infarct core are described in Supplementary material online, Results. Separate multivariable analyses were performed for (A) patient characteristics and angiographic data and (B) CMR data. Cardiac magnetic resonance parameters, which were all highly correlated with one another, were included separately in multiple stepwise regression models with patient characteristics and angiographic data to reduce multi-collinearity.

Similar results were obtained when area at risk, LV ejection fraction, LV end-systolic volume, and infarct size were included. Maximum leucocyte count (*P* = 0.053) and maximum monocyte count (*P* = 0.034) remained associates of infarct core native T1 after adjustment for LV end-diastolic volume. Similar results were also obtained in the multivariable model with LV end-diastolic volume for minimum leucocyte count (*P* = 0.011).

Infarct core native T1 (ms) was univariably associated with infarct core T2 (ms) (*r* = 0.42; *P* < 0.001).

Relationships for native T1 infarct core vs. infarct pathology, including infarct core T2, myocardial haemorrhage, and microvascular obstruction and 137 (86%) STEMI patients with a hypo-intense native T1 infarct core also had microvascular obstruction. In contrast, only 6.3% of those without hypo-intense infarct core had late microvascular obstruction. The negative- and positive predictive values of native T1 infarct core for T2 infarct core, myocardial haemorrhage, EGE, and microvascular obstruction are summarized in Supplementary material online, Table S2.

**Table 1:** Clinical and angiographic characteristics of 288 ST-elevation myocardial infarction patients who had cardiac magnetic resonance with evaluable maps for myocardial native T1 magnetization, including the subset of patients with an infarct core revealed by native T1 (all and categorized by tertiles of native T1)

Characteristics^a^	All patients (*n* = 288)	Patients with a native T1 infarct core (*n* = 160) (56%)	Patients with a native T1 infarct core grouped by tertile of infarct core zone native T1 (ms) at baseline			*P*-value
			T1 core ≤973 ms (*n* = 54) (33%)	974 <T1 core ≤1010 ms (*n* = 53) (33%)	T1 core >1010 ms (*n* = 53) (33%)	
Age (years)	59 (11)	59 (11)	59 (11)	57 (11)	61 (11)	0.238
Male sex, *n* (%)	211 (73)	123 (77)	46 (85)	37 (70)	40 (76)	0.144
BMI (kg/m^2^)	29 (5)	29 (5)	29 (4)	29 (5)	28 (5)	0.674
Medical history						
Hypertension, *n* (%)	93 (32)	57 (36)	17 (32)	21 (40)	19 (36)	0.684
Current smoking, *n* (%)	177 (62)	100 (62)	32 (59)	34 (64)	34 (64)	0.858
Hypercholesterolaemia, *n* (%)	82 (28)	44 (28)	12 (22)	17 (32)	15 (28)	0.527
Diabetes mellitus,^b^*n* (%)	32 (11)	20 (12)	7 (13)	7 (13)	6 (11)	1.000
Previous angina, *n* (%)	34 (12)	21 (13)	8 (15)	4 (8)	9 (17)	0.304
Previous myocardial infarction, *n* (%)	23 (8)	15 (9)	5 (9)	3 (6)	7 (13)	0.415
Previous PCI, *n* (%)	16 (6)	14 (9)	4 (7)	3 (6)	7 (13)	0.414
Presenting characteristics						
Heart rate (bpm)	78 (17)	78 (16)	80 (16)	79 (16)	76 (17)	0.401
Systolic blood pressure (mmHg)	136 (24)	136 (22)	137 (24)	140 (23)	131 (19)	0.095
Diastolic blood pressure (mmHg)	79 (14)	80 (14)	82 (14)	83 (13)	76 (13)	0.010
Time from symptom onset to reperfusion (min)	174 (120, 311)^a^	188 (125, 388)	223 (145, 406)	163 (113, 313)	198 (128, 257)	0.268
Ventricular fibrillation^c^, *n* (%)	20 (7)	150 (94)	3 (6)	2 (4)	5 (9)	0.518
Heart failure, Killip class at presentation, *n* (%)						
I	205 (71%)	101 (63)	29 (54%)	38 (72%)	34 (64%)	
II	64 (22%)	43 (27)	15 (28%)	14 (26%)	14 (26%)	0.059
III/IV	19 (7)	16 (10)	10 (18)	1 (2)	5 (9)	
ECG						
ST segment elevation resolution post-PCI, *n* (%)						
Complete, ≥70%	129 (45)	55 (35)	15 (28)	21 (40)	19 (36)	
Incomplete, 30% to <70%	115 (40)	74 (46)	27 (50)	23 (44)	24 (45)	0.715
None, ≤30%	43 (15)	30 (19)	12 (22)	8 (15)	10 (19)	
Reperfusion strategy, *n* (%)						
Primary PCI	268 (93)	148 (92)	49 (91)	49 (92)	50 (94)	
Rescue PCI (failed thrombolysis)	13 (4)	10 (6)	4 (7)	3 (6)	3 (6)	1.000
Successful thrombolysis	7 (2)	2 (1)	1 (2)	1 (2)	0 (0)	
Coronary angiography						
Number of diseased arteries,^d^*n* (%)						
1	156 (54)	89 (56)	156 (54)	156 (54)	156 (54)	
2	89 (29)	44 (28)	90 (31)	90 (31)	90 (31)	0.436
3	42 (15)	24 (15)	42 (15)	42 (15)	42 (15)	
LM	6 (2)	3 (2)	0 (0)	2 (4)	1 (2)	
Culprit artery, *n* (%)						
LAD	108 (38)	60 (38)	22 (41)	19 (36)	19 (36)	
LCX	51 (18)	31 (19)	10 (18)	12 (23)	9 (17)	0.915
RCA	129 (45)	69 (34)	22 (41)	22 (42)	25 (47)	
TIMI coronary flow grade pre-PCI, *n* (%)						
0/1	208 (72)	135 (84)	49 (91)	39 (74)	47 (89)	
2	52 (18)	27 (13)	5 (9)	11 (21)	5 (9)	0.085
3	28 (10)	4 (2)	0 (0)	3 (6)	1 (2)	
TIMI coronary flow grade post-PCI, *n* (%)						
0/1	3 (1)	2 (1)	0 (0)	1 (2)	1 (2)	
2	13 (4)	8 (5)	3 (6)	3 (6)	2 (4)	0.959
3	272 (94)	150 (94)	51 (94)	49 (92)	50 (94)	
Medical therapy						
ACE-I or ARB	285 (99)	159 (>99)	54 (100)	53 (100)	52 (98)	0.663
β-Blocker	278 (96)	158 (99)	53 (98)	52 (98)	53 (100)	1.000
Initial blood results on admission						
C-reactive protein, (mg/L), median (IQR), range	3.0 (2.0–7.0) 0–265.0	4.0 (2.0, 8.0) 1.0–265	3.5 (2.0–11.0) 1.0–125.0	3.0 (1.0–6.2) 1.0–92.0	4.0 (2.0–7.0) 1.0–265.0	0.696
Leucocyte cell count (×10^9^ L)	12.4 (3.5)	12.8 (3.6)	12.9 (3.5)	13.3 (3.5)	12.3 (3.6)	0.310
Neutrophil count (×10^9^ L)	9.6 (3.2)	10.1 (3.3)	10.0 (3.4)	10.6 (3.4)	9.6 (3.0)	0.244
Monocytes (×10^9^ L)	0.4 (0.4)	0.9 (0.4)	1.0 (0.4)	0.9 (0.3)	0.9 (0.5)	0.485
NT-proBNP (pg/mL)	824 (350, 1642)	1103 (628, 1849)	1456 (702, 2455)	980 (565, 1637)	1021 (529, 1436)	0.354

Missing data: heart rate, *n* = 1; time from symptom onset to reperfusion, *n* = 20; ST-segment resolution, *n* = 1; CRP, *n* = 7; leucocyte count, *n* = 1. The patients are grouped according to tertile of T1 in hypo-intense core at baseline.

ACE-I or ARB, angiotensin converting enzyme inhibitor or angiotensin receptor blocker; LAD, left anterior descending coronary artery; LCX, left circumflex coronary artery; LM, left main coronary artery; RCA, right coronary artery; TIMI, thrombolysis in myocardial infarction grade; PCI, percutaneous coronary intervention. Killip classification of heart failure after acute myocardial infarction: class I—no heart failure, class II—pulmonary rales or crepitations, a third heart sound, and elevated jugular venous pressure, class III—acute pulmonary oedema, and class IV—cardiogenic shock.

^a^Data are reported as mean (SD), median (IQR), or N (%) as appropriate. *P*-values have been obtained from a one-way ANOVA or Fisher test. Thrombolysis in myocardial infarction flow grades pre- and post-PCI were grouped 0/1 vs. 2/3 for this analysis.

^b^Successfully electrically cardioverted ventricular fibrillation at presentation or during emergency PCI procedure.

^c^Diabetes mellitus was defined as a history of diet-controlled or treated diabetes.

^d^Multi-vessel coronary artery disease was defined according to the number of stenoses of at least 50% of the reference vessel diameter, by visual assessment and whether or not there was left main stem involvement. The blood results on admission and their changes during the first 2 days after admission are described in Supplementary material online, Table S1.

**Table 2:** Comparison of cardiac magnetic resonance findings at baseline in 288 ST-elevation myocardial infarction survivors and 6-month cardiac magnetic resonance findings in 278 ST-elevation myocardial infarction patients

Characteristics*	All patients	Patients with a native T1 infarct core	Patients with a native T1 infarct core grouped by tertile of infarct core zone native T1 (ms) at baseline			*P*-value
	All patients (*n* = 288)	Hypo-intense core (*n* = 160)	≤973 ms (*n* = 54)	974 <T1 core ≤1014 ms (*n* = 53)	>1014 ms (*n* = 53)	
CMR findings 2 days post-MI						
LV ejection fraction (%)	55 (10)	52 (9)	52 (10)	51 (8)	53 (10)	0.418
LV end-diastolic volume, ml						
Men	162 (33)	168 (147, 187)	168 (22)	169 (36)	166 (30)	0.900
Women	124 (25)	125 (113, 145)	122 (30)	134 (26)	126 (21)	0.497
LV end-systolic volume (mL)						
Men	73 (55, 94)	79 (64, 98)	75 (64, 94)	81 (74, 103)	76 (60, 100)	0.496
Women	53 (41, 66)	64 (50, 69)	64 (57, 71)	66 (57, 70)	56 (45, 65)	0.383
LV mass (g)						
Men	142 (124, 159)	145 (130, 166)	149 (135, 170)	143 (126, 159)	141 (130, 160)	0.526
Women	97 (84, 108)	101 (89, 124)	103 (92, 113)	109 (93, 132)	97 (83, 101)	0.113
Oedema and infarct characteristics						
Area at risk, % LV mass	32 (12)	40 (11)	37 (11)	35 (10)	36 (11)	0.482
Infarct size, % LV mass	16 (7, 27)	25 (16, 32)	25 (18, 34)	27 (18, 32)	22 (16, 32)	0.386
Myocardial salvage, % of LV mass	18 (12, 24)	17 (12, 23)	18 (12, 24)	17 (10, 22)	16 (13, 22)	0.546
Myocardial salvage index, % of LV mass	62 (44, 84)	49 (36, 62)	50 (40, 62)	46 (30, 62)	50 (40, 63)	0.590
Late microvascular obstruction present, *n* (%)	145 (50)	23 (14)	49 (91)	45 (85)	43 (81)	0.356
Late microvascular obstruction, % LV mass	0.1 (0.0, 3.5)	2.7 (0.8, 7.5)	5.2 (1.7, 10.5)	2.7 (0.9, 7.1)	1.7 (0.3, 4.7)	0.005
Myocardial haemorrhage, *n* (%)*	96 (40)	94 (67)	34 (76)	35 (70)	25 (54)	0.086
Myocardial native T1 values						
T1 remote myocardium (all subjects) (ms)	961 (25)	964 (26)	958 (28)	962 (20)	972 (28)	0.014
Men	959 (25)	962 (26)	955 (29)	959 (19)	973 (26)	0.004
Women	968 (25)	969 (26)	969 (22)	969 (22)	968 (36)	0.992
T1 infarct zone (ms)	1097 (52)	1093 (52)	1052 (37)	1088 (33)	1140 (22)	<0.001
T1 hypo-intense infarct core (ms)	997 (57)	997 (57)	938 (30)	995 (12)	1060 (37)	<0.001
Myocardial native T2 values						
T2 infarct core (*n* = 171, ms)	54 (5)	54 (5)	52 (4)	53 (4)	56 (5)	<0.001
CMR findings 6 months post-MI (*n* = 267)						
LV ejection fraction at 6 months (%)	63 (57, 69)	60 (53, 65)	59 (53, 65)	59 (54, 64)	61 (54, 68)	0.542
LV end-diastolic volume at 6 months (mL)						
Men	165 (140, 193)	176 (155, 204)	188 (160, 209)	169 (153, 197)	171 (156, 196)	0.367
Women	124 (110, 136)	120 (96, 139)	120 (96, 139)	130 (122, 153)	127 (118, 142)	0.338
LV end-systolic volume at 6 months (mL)						
Men	61 (43, 78)	69 (56, 95)	73 (58, 98)	69 (62, 84)	63 (53, 96)	0.667
Women	43 (34, 58)	55 (44, 61)	45 (41, 56)	60 (50, 65)	53 (40, 57)	0.213

LV, left ventricle; T1, myocardial longitudinal relaxation time. Area at risk was measured with T2-mapping. Data are given as *n* (%) or mean (SD). *P*-values were obtained from one-way ANOVA, Kruskal–Wallis test, or a Fisher test. *Data are reported as mean (SD), median (IQR), or *n* (%) as appropriate. Data on T2*-CMR for myocardial haemorrhage were not available in 48 patients.

Three T1 maps (basal-, mid-, and distal-ventricular levels) were measured in each patient (*n* = 876 T1-maps overall). Overall, 20 (6.8%) patients had poor quality T1 maps and 4 (1.3%) patients had no evaluable T1 maps (*Figure 2*). In all, 42 (4.8%) T1 maps were unsuitable for analysis because of SSFP off-resonance artefacts and 19 (2.2%) T1 maps were affected by motion artefacts. T1 values were higher in infarct tissue surrounding the infarct core than within the infarct core (*P* < 0.001) and remote myocardium (*P* < 0.001).

### Infarct core tissue characteristics as a marker of subsequent left ventricular remodelling (Hypothesis 2)

At 6 months, LV end-diastolic volume increased on average (SD) by 5 (25) ml in 262 patients with evaluable data (*Table 2*). Adverse remodelling occurred in 30 (12%) patients and 23 (77%) of these patients had a hypo-intense native T1 core at baseline. Infarct core native T1 (ms) was not associated with change in LV end-diastolic volume at follow-up (*P* = 0.531). In multivariable regression, native T1 (ms, continuous) within the hypo-intense core was inversely associated with adverse remodelling (*Table 4*).

**Table 4:** Multivariable associates of adverse LV remodelling revealed by cardiac magnetic resonance in ST-elevation myocardial infarction survivors^a^ after 6 months follow-up

Multivariable associations	Odds ratio (95% CI)	*P*-value
A. Patient and angiographic characteristics		
Native T1 infarct core, per 10 ms	**0.91 (0.82, 1.00)**	**0**.**061**
Current smoking	5.27 (1.07, 26.00)	0.041
Sustained ventricular arrhythmia	16.06 (1.67, 154.43)	0.016
Incomplete ST-segment resolution	3.29 (0.85, 12.78)	0.085
B. Patient and angiographic characteristics and infarct core native T2		
Native T1 infarct core, per 10 ms	**0.91 (0.81, 1.01)**	**0**.**073**
Native T2 infarct core, per 10 ms	1.01 (0.28, 3.67)	0.987
Current smoking	4.99 (0.99, 25.06)	0.051
Sustained ventricular arrhythmia	15.26 (1.57, 148.71)	0.019
Incomplete ST-segment resolution	3.18 (0.81, 12.43)	0.097
C. Patient and angiographic characteristics and myocardial haemorrhage		
Native T1 infarct core, per 10 ms	**0.90 (0.81, 1.01)**	**0**.**070**
Myocardial haemorrhage	0.57 (0.14, 2.41)	0.449
Current smoking	4.78 (0.83, 27.52)	0.080
Sustained ventricular arrhythmia	11.70 (0.94, 144.88)	0.055
Incomplete ST-segment resolution	3.68 (0.90, 15.02)	0.069

The odds ratio (95% CIs) indicates the magnitude and direction for adverse LV remodelling. For a 10 ms increase in native T1, the odds ratio for adverse LV remodelling reduced (0.91 (0.82, 1.00); *P* = 0.061). For a 1 ms increase in native T1, the odds ratio for adverse LV remodelling reduced [0.99 (0.98, 1.00); *P* = 0.061].

^a^Twenty clinical characteristics at baseline that were univariable associates of adverse LV remodelling at 6 months post-MI were included in the multivariable model and these univariable associates are described in Supplementary material online, Results. Two hundred and sixty-seven STEMI patients had CMR at 6 months and baseline and 23 of these patients had missing data of at least one of the univariable characteristics that were included in this multivariable model. C-statistic [area-under-the-curve (AUC)] for the multivariable model in 244 subjects but not including native T1 core: 0.95; C-statistic (AUC) for the model (above) including infarct core native T1 (*n* = 136): 0.81; net reclassification index for incremental addition of T1 core to the model: 0.31, *P* = 0.184.

When the multivariable model for adverse remodelling included infarct size, the AUC without native T1 core (continuous, ms) was 0.823 and the AUC with T1 core values included was 0.857. Inclusion of native T1 core values neither increased nor reduced the predictive value of this model (net reclassification index *P* = 0.16).

There was no threshold for native T1 core value in the infarct core in relation to its association with LV outcomes at baseline or during follow-up.

In a sensitivity analysis, the occurrence of a hypo-intense core within the infarct zone on T1 mapping was associated with the odds ratio for being in the top quarter of an increase in LV end-diastolic volume at 6 months (native T1 core to predict Q4 (*n* = 66) vs. Q1–Q3 (*n* = 196) (*n* = 26 missing); odds ratio 0.994 (0.987, 0.999); *P* = 0.048).

#### Native T1 infarct core, microvascular obstruction, and left ventricular outcomes at 6 months

The relationships for infarct core native T1 (binary and continuous), T2 core (binary and continuous), microvascular obstruction (binary, % LV mass), and myocardial haemorrhage for LV outcomes, including LV end-diastolic volume and LV ejection fraction, are shown in *Table 5*. The presence of a hypo-intense infarct core disclosed by native T1 and native T2, the presence and amount of microvascular obstruction, and the occurrence of myocardial haemorrhage, were consistently associated with LV outcomes. Native T1 (ms) was not associated with LV volumes at follow-up, and there was no evidence of non-linearity between infarct core T1 (ms) and LV outcomes.

**Table 5:** The univariable relationships for infarct core characteristics revealed by native T1 and microvascular obstruction for LV outcomes at baseline and during follow-up in 288 ST-elevation myocardial infarction patients

		LVEDV at baseline	LVEDV at 6 months	LVEF at baseline	LVEF at follow-up
T1 core (per 10 ms)	Standardized β	−0.042	−0.035	0.151	0.055
	*P*-value	0.596	0.520	0.057	0.485
T1 core (binary)	*β*	16.410	13.80	−6.642	−4.652
	*P*-value	<0.0001	<0.0001	<0.0001	<0.0001
T2 core (per 10 ms)	Standardized *β*	0.035	0.057	0.159	−0.033
	*P*-value	0.653	0.282	0.037	0.586
T2 core (binary)	*β*	15.538	12.875	−6.542	−4.494
	*P*-value	<0.001	<0.0001	<0.0001	<0.0001
Myocardial haemorrhage (T2* core, binary)	*β*	17.205	16.811	−6.374	−5.769
	*P*-value	<0.0001	<0.0001	<0.0001	<0.0001
Microvascular obstruction (% of LV mass)	Standardized *β*	0.186	0.209	−0.443	−0.283
	*P*-value	0.002	<0.0001	<0.0001	0.004
Microvascular obstruction (binary)	*β*	15.853	12.454	−6.620	−4.464
	*P*-value	<0.001	<0.0001	<0.0001	<0.0001

The relationships for infarct core native T1 relaxation time (per 10 ms), native T1 infarct core (binary), and the presence and the amount of microvascular obstruction (*n* = 145 STEMI patients) with LV outcomes are summarized by *P*-values and, for continuous predictors, standardized regression coefficients or odds ratios per SD increase in native T1 (ms) or extent of microvascular obstruction (% of LV mass). Models with follow-up data are adjusted for baseline. Binary predictors are summarized by *P*-values and unstandardized regression coefficients or odds ratios (listed beneath table).

The odds ratio (*P*-values) for adverse remodelling and infarct core characteristics are: native T1 core (per 10 ms): 0.939, *P* = 0.122; native T1 core (present/absent): 2.692, *P* = 0.016; T2 core (present/absent): 2.874, *P* = 0.026; myocardial haemorrhage (present/absent): 2.556, *P* = 0.025; microvascular obstruction (% LV mass): 1.112, *P* = 0.004; microvascular obstruction (present/absent): 1.883, *P* = 0.115.

LVEDV, left ventricular end-diastolic volume; LVEF, left ventricular ejection fraction.

Infarct core native T1 early post-MI was associated with the concentration of NT-proBNP, a biochemical measure of LV remodelling, at 6 months independent of LV end-diastolic volume at baseline (Supplementary material online, Results).

### Infarct core tissue characteristics and health outcomes (Hypothesis 3)

All 288 patients had long-term follow-up data completed. Thirty (10.4%) patients died or experienced a heart failure event. These events included 5 cardiovascular deaths, 3 non-cardiovascular deaths, and 22 episodes of heart failure (Killip Class 3 or 4 heart failure (*n* = 20) or defibrillator implantation *n* = 2). Thirteen (4.5%) patients died or experienced a first heart failure hospitalization post-discharge, and 8 (61.5%) of these patients had a hypo-intense infarct core at baseline.

**Figure 3:**
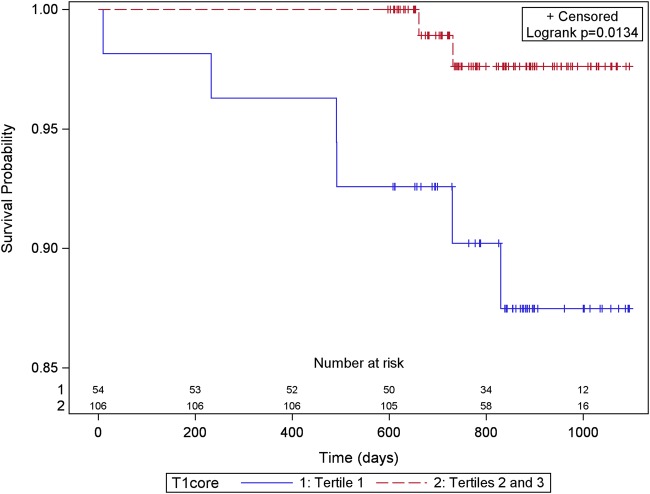
Kaplan–Meier survival curves for 160 ST-elevation myocardial infarction patients grouped according to the native T1 value in the infarct core with patients grouped by thirds (lowest T1 tertile vs. tertiles 2 and 3) and all-cause death or first heart failure hospitalization (*n* = 13) after discharge from hospital to the end of follow-up [censor time 839 (598–1099) days]. Infarct core native T1 values in the lowest tertile were associated with all-cause death or heart failure hospitalization.

Native T1 values (ms) within the hypo-intense infarct core (*n* = 160 STEMI patients) were inversely associated with the risk of all-cause death or first hospitalization for heart failure post-discharge (for a 10 ms increase in native T1: hazard ratio 0.730, 95% confidence interval (CI) 0.617, 0.863; *P* < 0.001) including after adjustment for LVEF at baseline, infarct core T2 (10 ms difference), and myocardial haemorrhage (*Figure 3*; *Table 6*). Infarct core T1 retained its prognostic significance over and above infarct core T2 and myocardial haemorrhage (*Table 6*, models C–F). The net reclassification index for the inclusion of infarct core native T1 (10 ms) in a multivariable prognostic model for all-cause death or heart failure post-discharge was 1.129 (95% CI 0.516, 1.742); *P* < 0.001) (*Table 6*). Using ROC analysis, the C-index for infarct core native T1 for all-cause death or heart failure was 0.806. The C-indexes for the prognostic model without and with infarct core native T1 (ms) were 0.715 and 0.931, respectively.

**Table 6:** Relationships for infarct core T1 and T2 relaxation times (10 ms) revealed by cardiac magnetic resonance at baseline in 160 ST-elevation myocardial infarction patients with an infarct core and all-cause death or first hospitalization for heart failure post-discharge [median (range) follow-up duration of 841 (723–945) days]

Associations	Hazard ratio (95% CI)	*P*-value
Univariable associations		
Infarct core native T1 (for a 10 ms difference)	**0.730 (0.617, 0.863)**	**<0**.**001**
Myocardial haemorrhage	2.488 (0.814, 7.609)	0.110
LVEF at baseline (for a 1% difference)	0.934 (0.885, 0.985)	0.013
Peak log eosinophil count (×10^9^/L)	0.617 (0.432, 0.881)	0.008
Model A		
Infarct core native T1 (for a 10 ms difference)	**0.744 (0.627, 0.883)**	**<0**.**001**
LVEF at baseline (1% difference)	0.938 (0.883, 0.996)	0.036
Model B		
Infarct core native T1 (for 10 ms difference)	**0.737 (0.621, 0.875)**	**<0**.**001**
Peak log eosinophil count (1 ×10^9^/L)	0.728 (0.476, 1.114)	0.144
Univariable associations		
Infarct core native T2 (for a 10 ms difference)	0.186 (0.032, 1.094)	0.063
Model C		
Infarct core native T2 (for a 10 ms difference)	**0.244 (0.039, 1.528)**	**0**.**132**
LVEF at baseline (for a 1% difference)	0.932 (0.870, 0.998)	0.044
Model D		
Infarct core native T2 (for 10 ms difference)	**0.203 (0.034, 1.297)**	**0**.**093**
Peak log eosinophil count (×10^9^/L)	0.681 (0.460, 1.007)	0.054
Model E		
Infarct core T1 (for 10 ms difference)	**0.738 (0.624, 0.873)**	**<0**.**001**
Myocardial haemorrhage	1.965 (0.229, 16.864)	0.538
Model F		
Infarct core T1 (for 10 ms difference)	**0.752 (0.634, 0.893)**	**0**.**001**
Infarct core T2 (for a 10 ms difference)	0.428 (0.068, 2.683)	0.365
Myocardial haemorrhage	1.485 (0.159, 13.879)	0.729

Thirteen (8.1%) patients experienced all-cause death or heart failure hospitalization post-discharge. Given the limited number of adverse events, the models were specified to assess the prognostic relationships of infarct core native T1 vs. circulating markers of systemic inflammation, LV function, LV volume, and infarct characteristics that were measured at approximately the same time 2 days after hospital admission.

LVEF, left ventricular ejection fraction. Infarct core T1 (10 ms difference) is highlighted in bold.

#### Prognostic importance of infarct core native T1: comparisons with microvascular obstruction and longer term health outcomes

In univariate Cox models for infarct core native T1 (ms), native T1 core (binary), T2 core (ms), T2 core (binary), myocardial haemorrhage and the presence (binary) and amount of microvascular obstruction (% LV mass), only infarct core native T1 (ms) (*P* < 0.001) and the amount of microvascular obstruction (% LV mass) (*P* < 0.001) were associated with all-cause death or first heart failure hospitalisation after discharge. In a post-hoc analysis stimulated by peer review, the odds ratio for infarct core T1 (10 ms) at baseline for the occurrence of all-cause death, heart failure hospitalisation or adverse LV remodelling was 0.92 (95% CI 0.85, 0.99), *P* = 0.0312. The inverse relationships between infarct core native T1 (ms) and LV surrogate and adverse health outcomes were reasonably linear, and there was no cut-off value for infarct core T1 (ms) for these outcomes.

## Discussion

The main findings of our study are (i) native T1 mapping revealed without an intravenous contrast agent resulted in evaluable scans in 96% of STEMI survivors 2 days post-MI; (ii) acute culprit coronary artery blood flow and circulating measures of systemic inflammation at the time of the hospital admission were multivariable associates of native T1 within the hypo-intense infarct core revealed by T1 mapping 2 days later; (iii) native T1 values (ms) within the infarct core were clinically meaningful since they were independently associated with adverse remodelling, NT-proBNP concentrations at 6 months, and all-cause death or heart failure hospitalization post-discharge during longer term follow-up; (iv) compared with infarct core T2 or myocardial haemorrhage revealed by T2* mapping, infarct core T1 was more consistently associated with LV surrogate outcomes and all-cause death or heart failure hospitalization (*Table 6*), implying T1 core is more closely linked with infarct pathology; (v) compared with microvascular obstruction revealed by contrast-enhanced CMR, a hypo-intense infarct core revealed by T1 mapping had similar prognostic significance for LV outcomes at 6 months and for post-discharge cardiac events including all-cause mortality and heart failure hospitalization in the longer term (*Tables 5* and *6*). Finally, our paper adds to the emerging literature on the prognostic value of quantitative native T1 CMR^39^ and reaffirms the prognostic importance of MVO post-STEMI.^3^

The results of this study extend what is known about infarct core pathology, and also provide a potential mechanistic explanation. Infarct size^1,2^ and pathology, including microvascular obstruction,^3^ haemorrhage,^5^ and salvage,^4^ predict cardiac morbidity and mortality post-MI. These pathologies are revealed by contrast-enhanced CMR, and until recently, the assessment of infarct tissue without an intravenous contrast agent has been limited to T2-weighted and T2* imaging of myocardial haemorrhage.^5,11,29,40,41^ T1-mapping methods, including MOLLI^15,20^ and shMOLLI,^42,43^ can now be integrated into clinical CMR protocols. Previous studies have assessed myocardial native T1 in experimental MI models *ex vivo*,^8,10^*in vivo*,^11^ or in proof-of-concept clinical studies involving much smaller numbers of MI patients.^9,12–15^ Our study extends these findings in a much larger STEMI cohort and provides new evidence that native T1 core is more reflective of the severity of infarct injury and its prognostic importance than infarct core T2 and potentially also myocardial haemorrhage.

We also compared infarct core pathology delineated by native T1 mapping with microvascular obstruction, which is an established prognostic CMR biomarker post-MI.^3^ Native T1 mapping is obtained without the use of an intravenous gadolinium-based contrast agent whereas microvascular obstruction is revealed by CMR imaging of EGE and LGE after intravenous contrast administration. We observed a high degree of concordance between the occurrence of a hypo-intense infarct core depicted by native T1 CMR (56%) and microvascular obstruction (50%) as revealed by contrast-enhanced CMR. Although both a native T1 core and microvascular obstruction are depicted as a hypo-intense core within the hyperintense infarct zone (*Figure 2*), the physics of the CMR techniques is entirely different. A hypo-intense infarct core depicted by non-contrast native T1 mapping is due to local destruction of the T1 magnetization signal. On the other hand, microvascular obstruction (*Figure 2*) is due to a failure of gadolinium contrast to penetrate within the infarct core. Both CMR methods are T1-weighted but contrast kinetics are not relevant for native T1 mapping since intravenous contrast is not administered. Accordingly, T1 mapping avoids the theoretical risks and actual restrictions associated with contrast-enhanced CMR. Furthermore, acquisition of the native T1 map does not prolong the CMR scan, in contrast to late gadolinium enhancement imaging for microvascular obstruction which is typically imaged 10–15 min after dosing.^19^

Culprit artery coronary flow at the end of emergency PCI reflects the efficacy of coronary reperfusion, and reduced coronary flow initially independently predicted native T1 relaxation time within infarct core as assessed by CMR 2 days later. Similar associations also exist for microvascular obstruction,^44,45^ and in our study, both infarct core native T1 and microvascular obstruction were independently associated with circulating biomarkers of acute systemic inflammation. The occurrence of an infarct core disclosed by native T1 mapping, and the nature of the core (i.e. the native T1 value), was associated with the initial severity of MI (i.e. Killip heart failure class), systemic inflammation (i.e. leucocyte counts), and LV remodelling and health outcomes in the longer term. We think that the prognostic significance of native T1 values within the hypo-intense core are a distinctive attribute compared with microvascular obstruction since signal-intensity values within microvascular obstruction are not clinically meaningful beyond binary categorization (i.e. present/absent).

### Limitations

We performed a single centre natural-history study involving near-consecutive STEMI admissions. The STEMI patients in our natural-history study were recruited 24/7 therefore flow cytometry and routine NT-proBNP testing in all participants was not pragmatically possible.

T1 assessment is sensitive to motion artefacts and imperfect breath holding, which and may reduce image quality. A shortened version of this sequence (ShMOLLI) involving only nine heart beats has been developed. This method shortens breath hold time and may help to account for these limitations.^42^ Despite this, the MOLLI method has high precision reproducibility. Our T1 measurements are in good agreement with *in vivo* data published in the literature, including previous measurements using the ShMOLLI sequence.^42,43^

The limited number of adverse events constrained the number of variables that could be included in the multivariable models (e.g. *Tables 4* and *6*); however, the associations between infarct core native T1 and a range of surrogate and clinical outcomes including adverse remodelling revealed by CMR, NT-proBNP, and the primary health outcome (all-cause death/heart failure), supports the adverse prognostic importance of infarct core native T1. Our study does not permit inference on causality, and other interpretations of our data are possible and further studies are warranted.

### Conclusions

We found that infarct core pathology revealed by native T1 maps had similar prognostic value compared with microvascular obstruction revealed by late gadolinium enhancement CMR. Native T1 mapping is potentially widely applicable in clinical practice, not limited by renal disease, and so potentially could represent an alternative non-contrast CMR option for the assessment of infarct pathology.

## Supplementary material

Supplementary material is available at *European Heart Journal* online.

## Authors’ contributions

C.H., I.F.: performed statistical analysis. C.B., K.O., I.F.: handled funding and supervision. D.C., M.M., M.P., H.E., M.L., S.W., S.H., A.M.: acquired the data. C.B., I.F.: conceived and designed the research. D.C., C.B., C.H.: drafted the manuscript. D.C., C.B., C.H., N.S., P.W.: made critical revision of the manuscript for key intellectual content. S.R., I.M., N.A., N.T., A.R.: have contributed other than the above listed.

## Funding

This research was supported by the British Heart Foundation Grant (Project Grant PG/11/2/28474), the National Health Service, and the Chief Scientist Office. Professor Berry was supported by a Senior Fellowship from the Scottish Funding Council. Dr Welsh is supported by BHF Fellowship FS/12/62/29889. Funding to pay the Open Access publication charges for this article was provided by the University of Glasgow.

**Conflict of interest:** This project was supported by research collaboration with Siemens Healthcare.

## Supplementary Material

Supplementary Data
