# Supplementary material for: Prognostic significance of infarct core pathology revealed by quantitative non-contrast in comparison with contrast cardiac magnetic resonance imaging in reperfused ST-elevation myocardial infarction survivors
Source: Eur Heart J. 2015 Aug 10;37(13):1044–59. doi: 10.1093/eurheartj/ehv372 (PMC4816961; doi:10.1093/eurheartj/ehv372)
Supplement: Supplementary Data [file ehv372_supplementary_data.zip › ehv372supp_data1.docx]

# Supplementary Methods

# Prognostic significance of infarct core pathology revealed by quantitative non-contrast in comparison to contrast cardiac magnetic resonance imaging in reperfused ST-elevation myocardial infarction survivors.

# ClinicalTrials.gov registration NCT02072850Table of contents

[Supplementary Methods 1](#_Toc420215543)

[Setting and study populations 3](#_Toc420215545)

[Coronary angiogram acquisition and analyses 4](#_Toc420215546)

[Percutaneous coronary intervention 4](#_Toc420215547)

[Angiographic analysis 5](#_Toc420215548)

[Outcome definitions 5](#_Toc420215549)

[CMR acquisition and analyses 6](#_Toc420215550)

[Electrocardiogram 10](#_Toc420215551)

[Biochemical and haematologic measurement of inflammation 11](#_Toc420215552)

[Hematologic measurement of inflammation 11](#_Toc420215553)

[Statistics 13](#_Toc420215554)

[Trial Management 15](#_Toc420215555)

[Health outcomes and their definitions 15](#_Toc420215556)

[References 16](#_Toc420215557)

[Clinical Event Adjudication Charter 21](#_Toc420215558)

[Objective of the Event Adjudication Charter 23](#_Toc420215562)

[Events to be reviewed by the independent cardiologist 23](#_Toc420215563)

[Adverse Event definitions 25](#_Toc420215564)

References………………………………………………………………………………… 53

# Setting and study populations

### STEMI patients

Screening, enrolment, and data collection were prospectively performed by cardiologists in the cardiac catheterization laboratories of the Golden Jubilee National Hospital, Glasgow, United Kingdom. This hospital is a regional referral centre for primary and rescue percutaneous coronary intervention (PCI). The hospital provides clinical services for a population of 2.2 million. A screening log was recorded, including patients who did not participate in the cohort study.

### Healthy volunteers

The purpose of including healthy volunteers was to collect normative reference data for myocardial native T1 in individuals without prior cardiovascular disease or therapy and who were reasonably representative of the population of individuals from whom the STEMI patients were drawn. Second, the reference native T1 values were required to be measured on the same CMR scanner and with the same protocol that was used for the STEMI patients including during the same time-period.

Healthy volunteers were invited to participate by placing adverts in public buildings (e.g. hospital, University) and through personal contacts of the researchers. Matching and selection of the healthy volunteers was done by the researchers in order to reflect the age and gender distribution of the STEMI patients. The healthy volunteers were resident in the same catchment area as the STEMI population. Fifty age- and gender-matched healthy volunteers who had a normal ECG and no prior history of cardiovascular disease or therapy underwent CMR during the same time period. The absence of late gadolinium enhancement (myocardial fibrosis or scar) was determined qualitatively by visual assessment, and the absence of late gadolinium enhancement was a requirement for inclusion of the volunteer in this analysis.

The rationale for including healthy volunteers in this study is as follows. First, native T1 values may vary between CMR scanners and so a local reference range for native T1 is recommended in CMR guidelines [1, 2]. Second, native T1 may vary spatially in the heart and therefore, since the focus of our study was to assess native T1 in myocardium remote from the infarct zone, we aimed to collect native T1 values in different segments of the heart in order to compare the remote zone native T1 values from STEMI patients with reference spatially matched remote zone native T1 values in age- and gender-matched healthy volunteers. Myocardial native T1 values were regionally segmented in regions-of-interest and summarised according to the AHA model [3].

# Coronary angiogram acquisition and analyses

Coronary angiograms were acquired during usual care with cardiac catheter laboratory X-ray (Innova®) and IT equipment (Centricity®) made by GE Healthcare. The coronary anatomy and disease characteristics of study participants were described based on the clinical reports of the attending cardiologist.

# Percutaneous coronary intervention

Consecutive admissions with acute ST-elevation myocardial infarction (STEM) referred for emergency percutaneous coronary intervention (PCI) were screened for the inclusion and exclusion criteria. During ambulance transfer to the hospital, the patients received 300 mg of aspirin, 600 mg of clopidogrel and 5000 IU of unfractionated heparin [4, 5]. The initial primary PCI procedure was performed using radial artery access. A conventional approach to primary PCI was adopted in line with usual care in our hospital [4, 5]. Conventional bare metal and drug eluting stents were used in line with guideline recommendations and clinical judgement. The standard transcatheter approach for reperfusion involves minimal intervention with aspiration thrombectomy only or minimal balloon angioplasty (e.g. a compliant balloon sized according to the reference vessel diameter and inflated at 4-6 atmospheres 1-2 times). During PCI, glycoprotein IIbIIIa inhibitor therapy was initiated with high dose tirofiban (25 μg/kg/bolus) followed by an intravenous infusion of 0.15 μg/kg/min for 12 hours, according to clinical judgement and indications for bail-out therapy [4, 5]. No reflow was treated according to contemporary standards of care with intra-coronary nitrate (i.e. 200 μg) and adenosine (i.e. 30 – 60 μg) [4, 5], as clinically appropriate. In patients with multivessel coronary disease, multivessel PCI was not recommended, in line with clinical guidelines [4, 5]. The subsequent management of these patients was symptom-guided.

# Angiographic analysis

The coronary anatomy and disease characteristics of study participants were described based on the clinical reports of the attending cardiologist.

# Outcome definitions

Coronary blood flow can be described based on the visual assessment of coronary blood flow revealed by contrast injection into the coronary arteries [4, 5].

| TIMI Coronary Flow Grade |  |
| --- | --- |
| 0 | No flow |
| 1 | Minimal flow past obstruction |
| 2 | Slow (but complete) filling and slow clearance |
| 3 | Normal flow and clearance |

# CMR acquisition and analyses

## CMR acquisition

CMR was performed on a Siemens MAGNETOM Avanto (Erlangen, Germany) 1.5-Tesla scanner with a 12-element phased array cardiac surface coil. T1 maps were acquired in 3 short-axial slices (basal, mid and apical), using an optimised modified look-locker inversion-recovery (MOLLI) investigational prototype sequence [6, 7] before contrast administration (Supplementary Methods). The MOLLI T1 cardiac-gated acquisition involved three inversion-recovery prepared look locker experiments combined within one protocol (3 (3) 3 (3) 5) [7]. The CMR parameters were: bandwidth ~1090 Hz/pixel; flip angle 35°; echo time (TE) 1.1 ms; T1 of first experiment 100 ms; TI increment 80 ms; matrix 192 x 124 pixels; spatial resolution 2.2 x 1.8 x 8.0 mm; slice thickness 8 mm; scan time 17 heartbeats .

T2 maps were acquired in contiguous short axis slices covering the whole ventricle, using an investigational prototype T2-prepared (T2P) TrueFisp sequence [8, 9]. Typical imaging parameters were: bandwidth ~947 Hz/pixel; flip angle 70°; T2 preparations: 0 ms, 24 ms, and 55 ms respectively; matrix 160 x 105 pixels; spatial resolution 2.6 x 2.1 x 8.0 mm; slice thickness 8 mm.

T2*-maps were obtained using an investigational prototype T2* map sequence acquired in 3 short-axis slices (basal, mid and apical). Typical imaging parameters were: bandwidth ~814 (x8) Hz/pixel; flip angle 18°; matrix 256x115; spatial resolution 2.6 x 1.6 x 10 mm; slice thickness 8 mm.

Late gadolinium enhancement images covering the entire LV were acquired 10-15 minutes after intravenous injection of 0.15 mmol/kg of gadoterate meglumine (Gd^2+^-DOTA, Dotarem, Guebert S.A.) using segmented phase-sensitive inversion recovery (PSIR) turbo fast low-angle shot [10]. Microvascular obstruction was defined as a dark zone on early delayed enhancement imaging 1, 3, 5 and 7 minutes post-contrast injection and within an area of late gadolinium enhancement. Typical imaging parameters were: matrix = 192 x 256, flip angle = 25°, TE = 3.36 ms, bandwidth = 130 Hz/pixel, echo spacing = 8.7ms and trigger pulse = 2. The voxel size was 1.8 x 1.3 x 8 mm^3^. Inversion times were individually adjusted to optimize nulling of apparently normal myocardium (typical values, 200 to 300 ms).

## MR image analyses

The images were analysed on a Siemens work-station by observers with at least 3 years CMR experience (N.A., D.C., I.M, S.R.). All of the images were reviewed by experienced CMR cardiologists (C.B., N.T.). LV dimensions, volumes and ejection fraction were quantified using computer assisted planimetry (syngo MR®, Siemens Healthcare, Erlangen, Germany). All scan acquisitions were spatially co-registered.

### T1 - standardized measurements in myocardial regions of interest

LV contours were delineated with computer assisted planimetry on the raw T1 image and copied onto the colour-encoded spatially co-registered maps. Apical segments were not included because of partial volume effects. Particular care was taken to delineate regions of interest with adequate margins of separation from tissue interfaces prone to partial volume averaging such as between myocardium and blood [1, 2, 11]. Each T1 map image was assessed for the presence of artefacts relating to susceptibility effects, or cardio-respiratory motion. Each motion-corrected series was evaluated for image alignment. Each map was evaluated against the original images. When artefacts occurred the affected segments were not included in the analysis.

In STEMI patients, myocardial T1 values were segmented spatially and regions-of-interest were defined as (1) remote myocardium, (2) injured myocardium and (3) infarct core. The regions-of-interest were planimetered to include the entire area of interest with distinct margins of separation from tissue interfaces to exclude partial volume averaging. The remote myocardial region-of-interest was defined as myocardium 180º from the affected zone with no visible evidence of infarction, oedema or wall motion abnormalities by inspecting corresponding contrast enhanced T1-weighted, T2-weighted and cine images, respectively. The infarct zone region-of-interest was defined as myocardium with pixel values (T1 or T2) >2 SD from remote myocardium on T2-weighted CMR [8, 9]. The infarct core was defined as an area in the centre of the infarct territory having a mean T1 value of at least 2 standard deviations below the T1 value of the periphery of the area-at-risk. A parameter was also created for native T1 in the infarct zone indexed to the remote zone.

In healthy volunteers, the mid-ventricular T1 map was segmented into 6 equal segments, using the anterior right ventricular-LV insertion point as the reference point [3]. T1 was measured in each of these segments, and regions-of-interest were planimetered distinct and separate from blood-pool and tissue interfaces. These segmental values were also averaged to provide one value per subject. Results are presented as average values for segments and slices.

### Infarct definition and size

The presence of acute infarction was established based on abnormalities in cine wall motion, rest first-pass myocardial perfusion, and delayed-enhancement imaging. In addition, supporting changes on the ECG and coronary angiogram were also required. Acute infarction was considered present only if late gadolinium enhancement was confirmed on both the axial and long axis acquisitions. The myocardial mass of late gadolinium (grams) was quantified using computer assisted planimetry and the territory of infarction was delineated using a signal intensity threshold of >5 standard deviations above a remote reference region and expressed as a percentage of total LV mass [12]. Infarct regions with evidence of microvascular obstruction were included within the infarct area and the area of microvascular obstruction was assessed separately and also expressed as a percentage of total LV mass.

### T2* – standardised measurements in myocardial regions of interest in serial imaging sub-study

LV contours were delineated with computer assisted planimetry on the raw T2* image and the last corresponding T2 raw image, with echo time of 55 ms [13]. Contours were then copied onto the colour-encoded spatially co-registered maps and corrected when necessary by consulting the SSFP cine images. Apical segments were not included because of partial volume effects. Particular care was taken to delineate regions of interest with adequate margins of separation from tissue interfaces prone to partial volume averaging such as between myocardium and blood. Each T2* map image was assessed for the presence of artefacts relating to susceptibility effects or cardio-respiratory motion. Each motion-corrected series was evaluated for image alignment. Each map was evaluated against the original images. When artefacts occurred, the affected segments were not included in the analysis.

### Myocardial haemorrhage

A hypointense core on T2* MRI was taken to represent myocardial haemorrhage.

### Area-at-risk

Area-at-risk was defined as LV myocardium with pixel values (T1/T2) >2 standard deviations from remote myocardium [8, 9, 14-17]. In order to assess the area-at-risk the epicardial and endocardial contours on the last corresponding T2-weighted raw image with an echo time of 55 ms were planimetered [13]. Contours were then copied to the map and corrected when necessary by consulting the SSFP cine images.

### Myocardial salvage

Myocardial salvage was calculated by subtraction of percent infarct size from percent area-at-risk [14, 17, 18]. The myocardial salvage index was calculated by dividing the myocardial salvage area by the initial area-at-risk.

### Adverse remodelling

Adverse remodelling was defined as an increase in LV end-diastolic volume ≥ 20% at 6 months from baseline [19].

### Reference ranges

Reference ranges used in the laboratory were 105 – 215 g for LV mass in men, 70 – 170 g for LV mass in women, 77 – 195 ml for LV end-diastolic volume in men, 52 – 141 ml for LV end-diastolic volume in women, 19 – 72 ml for LV end-systolic volume in men and 13 – 51 ml for LV end-systolic volume in women.

# Electrocardiogram

A 12 lead electrocardiogram (ECG) was obtained before coronary reperfusion and 60 minutes afterwards with Mac-Lab® technology (GE Healthcare) in the catheter laboratory and a MAC 5500 HD recorder (GE Healthcare) in the Coronary Care Unit. The ECGs were acquired by trained cardiology staff. The ECGs were de-identified and transferred to the local ECG management system. The ECGs were then analysed by the University of Glasgow ECG Core Laboratory which is certified to ISO 9001: 2008 standards as a UKAS Accredited Organization.

The extent of ST-segment resolution on the ECG assessed 60 minutes after reperfusion compared to the baseline ECG before reperfusion [4] was expressed as complete (≥70%), incomplete (30% to < 70%) or none (≤30%).

# Biochemical and hematologic measurement of inflammation

Serial systemic blood sample were obtained immediately after reperfusion in the cardiac catheterization laboratory, and subsequently between 0600 - 0700 hrs each day during the initial in-patient stay in the Coronary Care Unit. C-reactive protein (CRP) was measured in an NHS hospital biochemistry laboratory using a particle enhanced immunoturbimetric assay method (Cobas C501, Roche),) and the manufacturers calibrators and quality control material, as a biochemical measure of inflammation. The high sensitive assay CRP measuring range is 0.1-250 mg/L. The expected CRP values in a healthy adult are < 5 mg/L, and the reference range in our hospital is 0 - 10 mg/L. A blood sample was routinely obtained in the cardiac catheter laboratory immediately following revascularization and then again at 0700 hrs on the first and second days after admission to hospital.

NT-proBNP, a biochemical measure of LV wall stress, was measured in a research laboratory using an electrochemiluminescence method (e411, Roche) and the manufacturers calibrators and quality control material. The limit of detection is 5 pg/ml. Long-term coefficient of variations of low and high controls are typically <5%, and were all within the manufacturers range.

# Hematologic measurement of inflammation

Leucocyte count and leucocyte sub-populations were measured as a hematologic measure of inflammation using sheath flow technology incorporating semi-conductor laser beam, forward and side scattered light (Sysmex XT200i and XT1800i for white blood cell and differential white blood cell counts, respectively). The linearity ranges for white blood cells was 0.00-440.0 x10(9) /L. The following are the normal ranges for full blood count parameters:

|  | **MALE** | **FEMALE** |
| --- | --- | --- |
| WBC x 10^9/L | 4.0 - 11.0 | 4.0 - 11.0 |
| RBC x 10^12/L | 4.50 - 6.50 | 3.80 - 5.80 |
| Hgb g/L | 130 - 180 | 115 - 165 |
| HCT L/L | 0.400 - 0.540 | 0.370 - 0.470 |
| MCV fL | 78 - 99 | 78 - 99 |
| MCH Pg | 27.0 - 32.0 | 27.0 - 32.0 |
| MCHC g/L | 310 - 360 | 310 - 360 |
| PLATELETS x 10^9/L | 150 - 400 | 150 - 400 |
| NEUTROPHILS x 10^9/L | 2.5 - 7.5 | 2.5 - 7.5 |
| LYMPHOCYTES x 10^9/L | 1.5 - 4.0 | 1.5 - 4.0 |
| MONOCYTES x 10^9/L | 0.2 - 0.8 | 0.2 - 0.8 |
| EOSINOPHILS x 10^9/L | 0.0 - 0.4 | 0.0 - 0.4 |
| BASOPHILS x 10^9/L | 0.01 - 0.10 | 0.01 - 0.10 |

A blood sample was routinely obtained in the cardiac catheter laboratory, immediately following revascularization and then again at 0700 on the first and second days after admission to hospital.

# Statistics

### Sample size calculation

The sample size of 300 was predetermined based on the incidence of infarct pathology (e.g. myocardial haemorrhage or microvascular obstruction) affecting at least one third of the cohort. With an estimated haemorrhage incidence of 33% at 48 h post-STEMI, 100 subjects would have evidence of myocardial haemorrhage and 200 subjects would not. The study would have 90% power at a 5% level of significance using a two sided two sample t-test to detect a between-group difference in mean LV end-systolic volume index of 4.65 ml/m2 equivalent to three eighths of a common standard deviation (or an effect size of 0.375). We predicted a between-group difference in mean LVESVI of 4.65 ml/m2 equivalent to three eighths of a common standard deviation (or an effect size of 0.375). We also estimated that at least 30 MACE events would occur based on a conservative estimate of the event rate (10-12%) at 18 months. The sample size calculation was performed using nQuery version 7.0.

Categorical variables are expressed as number and percentage of patients. Most continuous variables followed a normal distribution and are therefore presented as means together with standard deviation. Those variables that did not follow a normal distribution are presented as medians with interquartile range. Differences in continuous variables between groups were assessed by the Student’s t-test or analysis of variance (ANOVA) for continuous data with normal distribution, otherwise the nonparametric Wilcoxon rank sum test or Kruskal-Wallis test. Differences in categorical variables between groups were assessed using a Chi-square test or Fisher’s test, as appropriate. Correlation analyses were Pearson or Spearman tests, as indicated. Random effects models were used to compute inter-and intra- rater reliability measures (intra-class correlation coefficient (ICC)) for the reliability of infarct core native T1 values measured independently by 2 observers in 12 randomly selected patients from the cohort.

Univariable and multivariable linear regression analyses were performed to identify associates of T1 values for (1) remote myocardium, (2) injured myocardium within the area-at-risk and (3) infarct core in all patients and (4) in patients without late microvascular obstruction. In backward stepwise linear regressions, the Akaike information criteria (AIC) was used as a measure of the relative quality of the models for this dataset, and the model with the minimum AIC value was reported. Where multicollinearity was problematic, analyses were performed with appropriate non-correlated subsets of variables. Where standardised regression coefficients are reported, these are calculated by multiplying the unstandardized coefficient by the standard deviation of the predictor, then dividing by the standard deviation of the response. Potential non-linear relationships between T1 values in regions of interest and LV ejection fraction and end-diastolic volume were explored with restricted cubic splines and Loess plots. The relationships between the presence or absence of an infarct core revealed by native T1 CMR compared with early MVO, late MVO, presence of T2 core, and myocardial haemorrhage were explored in sensitivity analyses.

Receiver operating curve (ROC), Kaplan-Meier and Cox proportional hazards methods were used to identify potential clinical predictors of all-cause death/heart failure events and MACE, including patient characteristics, CMR findings and native T1. The net reclassification improvement (NRI) was calculated as described by Pencina et al [^20^].

All p-values are 2-sided, and a p-value > 0.05 indicates the absence of a statistically significant effect. Statistical analyses were performed using R version 2.15.1 or SAS v 9.3, or higher versions of these programs.

# **Trial Management**

The study was conducted in line with Guidelines for Good Clinical Practice (GCP) in Clinical Trials. <http://www.mrc.ac.uk/documents/pdf/good-clinical-practice-in-clinical-trials/>

Trial management included a Trial Management Group, and an independent Clinical Trials Unit. Day to day study activity was coordinated by the Trial Management Group who was responsible to the Sponsor which was responsible for overall governance and that the trial was conducted according to GCP standards.

Clinical events were assessed and validated by an independent cardiologist (A.M.) who had access to relevant source clinical data. This cardiologist followed an agreed charter and he was blinded to all of the other clinical data.

# Health outcomes and their definitions

A comprehensive definition of adverse events [21, 22] and their adjudication is detailed in the Clinical Event Committee Charter.

We pre-specified adverse health outcomes that are pathophysiologically linked with STEMI. The primary composite outcome was (1) major adverse cardiac events (MACE) defined as cardiac death, non-fatal myocardial infarction (MI) or hospitalization for heart failure (Supplementary Methods). All-cause death or heart failure hospitalization was a secondary outcome.

After enrolment, research staff screened for MACE by checking the national electronic medical records of the study participants and by contacting the patients and their primary and secondary care physicians, as appropriate. Each event was reviewed by a cardiologist who was independent of the research team and blinded to all of the clinical and CMR data. The adverse events were defined according to standard guidelines and categorized as having occurred during the index admission or post-discharge. All study participants were followed-up for a minimum of 18 months after discharge.

# References

1. Kramer CM, Barkhausen J, Flamm SD, Kim RJ and Nagel E. Standardized cardiovascular magnetic resonance (CMR) protocols 2013 update. *Journal of cardiovascular magnetic resonance : official journal of the Society for Cardiovascular Magnetic Resonance*. 2013;15:91.

2. Moon JC, Messroghli DR, Kellman P, Piechnik SK, Robson MD, Ugander M, Gatehouse PD, Arai AE, Friedrich MG, Neubauer S, Schulz-Menger J, Schelbert EB, Society for Cardiovascular Magnetic Resonance I and Cardiovascular Magnetic Resonance Working Group of the European Society of C. Myocardial T1 mapping and extracellular volume quantification: a Society for Cardiovascular Magnetic Resonance (SCMR) and CMR Working Group of the European Society of Cardiology consensus statement. *Journal of cardiovascular magnetic resonance : official journal of the Society for Cardiovascular Magnetic Resonance*. 2013;15:92.

3. Cerqueira MD, Weissman NJ, Dilsizian V, Jacobs AK, Kaul S, Laskey WK, Pennell DJ, Rumberger JA, Ryan T and Verani MS. Standardized myocardial segmentation and nomenclature for tomographic imaging of the heart. A statement for healthcare professionals from the Cardiac Imaging Committee of the Council on Clinical Cardiology of the American Heart Association. *Circulation*. 2002;105:539-42.

4. Steg PG, James SK, Atar D, Badano LP, Blomstrom-Lundqvist C, Borger MA, Di Mario C, Dickstein K, Ducrocq G, Fernandez-Aviles F, Gershlick AH, Giannuzzi P, Halvorsen S, Huber K, Juni P, Kastrati A, Knuuti J, Lenzen MJ, Mahaffey KW, Valgimigli M, van 't Hof A, Widimsky P and Zahger D. ESC Guidelines for the management of acute myocardial infarction in patients presenting with ST-segment elevation. *European heart journal*. 2012;33:2569-619.

5. Windecker S, Kolh P, Alfonso F, Collet JP, Cremer J, Falk V, Filippatos G, Hamm C, Head SJ, Juni P, Kappetein AP, Kastrati A, Knuuti J, Landmesser U, Laufer G, Neumann FJ, Richter DJ, Schauerte P, Sousa Uva M, Stefanini GG, Taggart DP, Torracca L, Valgimigli M, Wijns W and Witkowski A. 2014 ESC/EACTS Guidelines on myocardial revascularization: The Task Force on Myocardial Revascularization of the European Society of Cardiology (ESC) and the European Association for Cardio-Thoracic Surgery (EACTS)Developed with the special contribution of the European Association of Percutaneous Cardiovascular Interventions (EAPCI). *European heart journal*. 2014;35:2541-619.

6. Messroghli DR, Radjenovic A, Kozerke S, Higgins DM, Sivananthan MU and Ridgway JP. Modified Look-Locker inversion recovery (MOLLI) for high-resolution T1 mapping of the heart. *Magnetic resonance in medicine : official journal of the Society of Magnetic Resonance in Medicine / Society of Magnetic Resonance in Medicine*. 2004;52:141-6.

7. Messroghli DR, Walters K, Plein S, Sparrow P, Friedrich MG, Ridgway JP and Sivananthan MU. Myocardial T1 mapping: application to patients with acute and chronic myocardial infarction. *Magnetic resonance in medicine : official journal of the Society of Magnetic Resonance in Medicine / Society of Magnetic Resonance in Medicine*. 2007;58:34-40.

8. Giri S, Chung YC, Merchant A, Mihai G, Rajagopalan S, Raman SV and Simonetti OP. T2 quantification for improved detection of myocardial oedema. *Journal of cardiovascular magnetic resonance : official journal of the Society for Cardiovascular Magnetic Resonance*. 2009;11:56.

9. Verhaert D, Thavendiranathan P, Giri S, Mihai G, Rajagopalan S, Simonetti OP and Raman SV. Direct T2 quantification of myocardial oedema in acute ischemic injury. *JACC Cardiovascular imaging*. 2011;4:269-78.

10. Kellman P, Arai AE, McVeigh ER and Aletras AH. Phase-sensitive inversion recovery for detecting myocardial infarction using gadolinium-delayed hyperenhancement. *Magnetic resonance in medicine : official journal of the Society of Magnetic Resonance in Medicine / Society of Magnetic Resonance in Medicine*. 2002;47:372-83.

11. Xue H, Greiser A, Zuehlsdorff S, Jolly MP, Guehring J, Arai AE and Kellman P. Phase-sensitive inversion recovery for myocardial T1 mapping with motion correction and parametric fitting. *Magnetic resonance in medicine : official journal of the Society of Magnetic Resonance in Medicine / Society of Magnetic Resonance in Medicine*. 2013;69:1408-20.

12. Flett AS, Hasleton J, Cook C, Hausenloy D, Quarta G, Ariti C, Muthurangu V and Moon JC. Evaluation of techniques for the quantification of myocardial scar of differing etiology using cardiac magnetic resonance. *JACC Cardiovascular imaging*. 2011;4:150-6.

13. Wassmuth R, Prothmann M, Utz W, Dieringer M, von Knobelsdorff-Brenkenhoff F, Greiser A and Schulz-Menger J. Variability and homogeneity of cardiovascular magnetic resonance myocardial T2-mapping in volunteers compared to patients with oedema. *Journal of cardiovascular magnetic resonance : official journal of the Society for Cardiovascular Magnetic Resonance*. 2013;15:27.

14. Eitel I, Desch S, Fuernau G, Hildebrand L, Gutberlet M, Schuler G and Thiele H. Prognostic significance and determinants of myocardial salvage assessed by cardiovascular magnetic resonance in acute reperfused myocardial infarction. *Journal of the American College of Cardiology*. 2010;55:2470-9.

15. Berry C, Kellman P, Mancini C, Chen MY, Bandettini WP, Lowrey T, Hsu LY, Aletras AH and Arai AE. Magnetic resonance imaging delineates the ischemic area at risk and myocardial salvage in patients with acute myocardial infarction. *Circulation Cardiovascular imaging*. 2010;3:527-35.

16. Payne AR, Casey M, McClure J, McGeoch R, Murphy A, Woodward R, Saul A, Bi X, Zuehlsdorff S, Oldroyd KG, Tzemos N and Berry C. Bright-blood T2-weighted MRI has higher diagnostic accuracy than dark-blood short tau inversion recovery MRI for detection of acute myocardial infarction and for assessment of the ischemic area at risk and myocardial salvage. *Circulation Cardiovascular imaging*. 2011;4:210-9.

17. Francone M, Bucciarelli-Ducci C, Carbone I, Canali E, Scardala R, Calabrese FA, Sardella G, Mancone M, Catalano C, Fedele F, Passariello R, Bogaert J and Agati L. Impact of primary coronary angioplasty delay on myocardial salvage, infarct size, and microvascular damage in patients with ST-segment elevation myocardial infarction: insight from cardiovascular magnetic resonance. *Journal of the American College of Cardiology*. 2009;54:2145-53.

18. Payne AR, Berry C, Doolin O, McEntegart M, Petrie MC, Lindsay MM, Hood S, Carrick D, Tzemos N, Weale P, McComb C, Foster J, Ford I and Oldroyd KG. Microvascular Resistance Predicts Myocardial Salvage and Infarct Characteristics in ST-Elevation Myocardial Infarction. *Journal of the American Heart Association*. 2012;1:e002246.

19. van Kranenburg M, Magro M, Thiele H, de Waha S, Eitel I, Cochet A, Cottin Y, Atar D, Buser P, Wu E, Lee D, Bodi V, Klug G, Metzler B, Delewi R, Bernhardt P, Rottbauer W, Boersma E, Zijlstra F and van Geuns RJ. Prognostic value of microvascular obstruction and infarct size, as measured by CMR in STEMI patients. *JACC Cardiovascular imaging*. 2014;7:930-9.

20. Thygesen K, Alpert JS, Jaffe AS, Simoons ML, Chaitman BR and White HD. Third universal definition of myocardial infarction. *Global heart*. 2012;7:275-95.

21. Karen A. Hicks HMJH, Kenneth W. Mahaffey, Roxana Mehran, Steven E. Nissen, Norman L. Stockbridge, Shari L. Targum, Robert Temple. On behalf of the Standardized Data Collection for Cardiovascular Trials Initiative Standardized Definitions for End Point Events in Cardiovascular Trials. <http://www.cdisc.org/stuff/contentmgr/files/0/2356ae38ac190ab8ca4ae0b222392b37/misc/cdisc_november_16__2010.pdf>

# Clinical Event Adjudication Charter

# Detection and Significance of Heart Injury in ST Elevation Myocardial Infarction –

# The BHF MR-MI study

#

# NCT02072850

## Rationale for the independent adjudication of clinical events

As a measure of enhanced Pharmacovigilance (PV) and Good Clinical Practice, a cardiologist who was independent of the clinical research team was designated to review deaths (due to any cause) and specifically cardiovascular events of interest. At a high level, such events of interest will include death of any cause, non-fatal acute myocardial infarction, non-fatal stroke, hospitalization due to unstable angina, hospitalization due to heart failure and coronary revascularization procedures (i.e. percutaneous coronary intervention, coronary artery bypass grafting). The revascularization procedures will not be considered to be major adverse events of interest but will be reviewed by the independent clinician to ensure that events of interest (e.g. acute myocardial infarction) have not been missed.

The clinician will review cases of interest to determine if they meet accepted diagnostic criteria. Causality assessments will not be made by the clinician, nor will the clinician possess governance authority. The cardiologist will be blinded regarding any information relating to the imaging measurements.

All deaths and pre-specified major adverse cardiovascular events (i.e. “MACE”-type events) will be prospectively collected by investigators and classified independently by the independent cardiologist. Details on these pre-specified events are listed in section 4.

As noted above, events of interest will be identified primarily by the investigator, who may use an eCRF checkbox to mark any event as a “CV event of interest”. The study was under regulatory review by the National Research Ethics Service and the National Waiting Times Board (NWTB) which is the Sponsor.

# Objective of the Event Adjudication Charter

The purpose of this document is to delineate the roles, responsibilities and procedures in regards to the adjudication of cardiovascular events occurring in the BHF MR-MI study.

## Study Coordinator

The independent cardiologist is assisted by the study coordinator (Dr David Carrick, BHF Cardiovascular Research Centre, University of Glasgow; [david.carrick@nhs.net](mailto:david.carrick@nhs.net)) who is a registered physician based in the University of Glasgow and Golden Jubilee National Hospital and who has considerable previous experience in the conduct of clinical cardiology studies.

The coordinator will:

Assist with preparation of the source clinical data

Enter the classification verdicts of the independent cardiologist into the database

# Events to be reviewed by the independent cardiologist

## 3.1 Deaths

The independent cardiologist will review all reported deaths and classify the cause of death according to the following schema:

Non-cardiovascular

A definite non-cardiovascular cause of death must be identified.

Cardiovascular (CV)

Death due to acute myocardial infarction

Death due to stroke

Sudden cardiac death

Other CV death (e.g. heart failure, pulmonary embolism, cardiovascular procedure-related)

Undetermined cause of death (i.e. cause of death unknown)

## 3.2 Non-fatal cardiovascular events

The independent cardiologist will review and adjudicate the following reported non-fatal cardiovascular events:

Acute myocardial infarction

Hospitalization for unstable angina/other angina*/chest pain*

Stroke/TIA/Other cerebrovascular events (i.e. subdural/extradural hemorrhage)**

Heart failure requiring hospitalization

Coronary revascularization procedures (i.e. percutaneous coronary intervention, coronary artery bypass grafting)***

Renal failure (>25% rise in creatinine from baseline or an absolute increase in serum creatinine of 0·.5 mg/dL (44 µmol/L) after a radiographic examination using a contrast agent (Barrett NEJM 2006;354:379-86)

Bleeding according to the ACUITY criteria (Stone Am Heart J 2004;148:764-75)

Note: Other non-fatal cardiovascular events will not routinely be reviewed by the independent cardiologist. These events will be reviewed by trained and qualified clinical research staff in the Golden Jubilee National Hospital to ensure that potential cardiovascular events requiring adjudication are not missed. If the review suggests that a potential cardiovascular event requiring adjudication may have been missed, further information will be requested, as required and, if necessary, the event will be allocated for adjudication.

*Hospitalization for other angina or for chest pain are not study events of interest but such events will be reviewed by the independent cardiologist to ensure that acute myocardial infarction or hospitalization for unstable angina events have not been missed.

**TIAs and other cerebrovascular events (subdural haemorrhage, extradural haemorrhage) will be reviewed to ensure that stroke events have not been missed.

***Coronary revascularization procedures (i.e. percutaneous coronary intervention, coronary artery bypass grafting) are not study events of interest but will be reviewed by the independent cardiologist to sure that study events of interest (e.g. acute myocardial infarction, hospitalization for unstable angina) have not been missed.

# Adverse Event definitions

For those event-types requiring adjudication, each event will usually be adjudicated on the basis of strict application of the endpoint definitions below. However, the clinical likelihood that a suspected event has occurred will be individually assessed even in the absence of fulfilment of all of the criteria specified in the event-definition, recognizing that information may at times be difficult to interpret (e.g. the exact measurement of ECG changes may be imprecise) or unavailable.

Overall, event definitions should align with the "Standardized definitions for endpoint events in cardiovascular trials' Hicks KA et al May 2011 and the "Third Universal Definition of Myocardial Infarction" Thygesen et al Eur Heart J 2012.

## 4.1 Deaths

In cases where a patient experiences an event and later dies due to that event, the event causing death and the death will be considered as separate events *only* if they are separated by a change in calendar day. If the event causing death and the death occur on the same calendar day, death will be the only event classified.

## 4.1.1 Cardiovascular deaths

**Cardiovascular death** includes death resulting from an acute myocardial infarction, sudden cardiac death, death due to heart failure, death due to stroke and death due to other cardiovascular causes as follows:

**Death due to Acute Myocardial Infarction** refers to a death usually occurring up to 30 days after a documented acute myocardial infarction (verified either by the diagnostic criteria outlined below for acute myocardial infarction, above, or by autopsy findings showing recent myocardial infarction or recent coronary thrombus) due to the myocardial infarction or its immediate consequences (e.g. progressive heart failure) and where there is no conclusive evidence of another cause of death.

If death occurs before biochemical confirmation of myocardial necrosis can be obtained, adjudication should be based on clinical presentation and other (e.g. ECG, angiographic, autopsy) evidence.

NOTE: This category will include sudden cardiac death, involving cardiac arrest, often with symptoms suggestive of myocardial ischemia, and accompanied by presumably new ST elevation*, or new left bundle branch block*, or evidence of fresh thrombus in a coronary artery by coronary angiography and/or at autopsy, but death occurring before blood samples could be obtained, or at a time before the appearance of cardiac biomarkers in the blood (i.e. myocardial infarction Type 3 – see section 4.2.1, below).

*If ECG tracings are not available for review, the independent cardiologist may adjudicate on the basis of reported new ECG changes that have been clearly documented in the case records or in the case report form.

Death resulting from a procedure to treat an acute myocardial infarction [percutaneous coronary intervention (PCI), coronary artery bypass graft surgery (CABG)], or to treat a complication resulting from acute myocardial infarction, should also be considered death due to acute myocardial infarction.

Death resulting from a procedure to treat myocardial ischemia (angina) or death due to an acute myocardial infarction that occurs as a direct consequence of a cardiovascular investigation/procedure/operation that was not undertaken to treat an acute myocardial infarction or its complications should be considered as a death due to other cardiovascular causes.

**Sudden Cardiac Death** refers to a death that occurs unexpectedly in a previously stable patient. The cause of death should not be due to another adjudicated cause (e.g. acute myocardial infarction Type 3 – see section 4.2.1 below).

The following deaths should be included.

a. Death witnessed and instantaneous without new or worsening symptoms

b. Death witnessed within 60 minutes of the onset of new or worsening symptoms unless a cause other than cardiac is obvious.

c. Death witnessed and attributed to an identified arrhythmia (e.g., captured on an ECG recording, witnessed on a monitor), or unwitnessed but found on implantable cardioverter-defibrillator review.

d. Death in patients resuscitated from cardiac arrest in the absence of pre-existing circulatory failure or other causes of death, including acute myocardial infarction, and who die (without identification of a non-cardiac aetiology) within 72 hours or without gaining consciousness; similar patients who died during an attempted resuscitation.

Unwitnessed death without any other cause of death identified (information regarding the patient’s clinical status in the 24 hours preceding death should be provided, if available)

**Death due to Heart Failure** refers to a death occurring in the context of clinically worsening symptoms and/or signs of heart failure without evidence of another cause of death (e.g. acute myocardial infarction).

Death due to heart failure should include sudden death occurring during an admission for worsening heart failure as well as death from progressive heart failure or cardiogenic shock following implantation of a mechanical assist device.

New or worsening signs and/or symptoms of heart failure include any of the following:

**a**. New or increasing symptoms and/or signs of heart failure requiring the initiation of, or an increase in, treatment directed at heart failure or occurring in a patient already receiving maximal therapy for heart failure

Note: If time does not allow for the initiation of, or an increase in, treatment directed at heart failure or if the circumstances were such that doing so would have been inappropriate (e.g. patient refusal), the adjudication will be based on the clinical presentation and, if available, investigative evidence.

**b**. Heart failure symptoms or signs requiring continuous intravenous therapy (i.e. at least once daily bolus administration or continuous maintenance infusion) or chronic oxygen administration for hypoxia due to pulmonary oedema.

**c**. Confinement to bed predominantly due to heart failure symptoms.

**d**. Pulmonary oedema sufficient to cause tachypnoea and distress **not** occurring in the context of an acute myocardial infarction, worsening renal function (that is not wholly explained by worsening heart failure/cardiac function) or as the consequence of an arrhythmia occurring in the absence of worsening heart failure.

**e**. Cardiogenic shock **not** occurring in the context of an acute myocardial infarction or as the consequence of an arrhythmia occurring in the absence of worsening heart failure.

Cardiogenic shock is defined as systolic blood pressure (SBP) < 90 mm Hg for greater than 1 hour, not responsive to fluid resuscitation and/or heart rate correction, and felt to be secondary to cardiac dysfunction and associated with at least one of the following signs of hypoperfusion:

Cool, clammy skin ***or***

Oliguria (urine output < 30 mL/hour) ***or***

Altered sensorium ***or***

Cardiac index < 2·2 L/min/m^2^

Cardiogenic shock can also be defined if SBP < 90 mm Hg and increases to ≥ 90 mm Hg in less than 1 hour with positive inotropic or vasopressor agents alone and/or with mechanical support.

**Death due to Stroke** refers to death after a documented stroke (verified by the diagnostic criteria outlined below for stroke or by typical post mortem findings) that is either a direct consequence of the stroke or a complication of the stroke and where there is no conclusive evidence of another cause of death.

NOTE: In cases of early death where confirmation of the diagnosis cannot be obtained, the independent may adjudicate based on clinical presentation alone.

Death due to a stroke reported to occur as a direct consequence of a cardiovascular investigation/procedure/operation will be classified as death due to other cardiovascular cause.

Death due to subdural or extradural haemorrhages will be adjudicated (based on clinical signs and symptoms as well as neuroimaging and/or autopsy) and classified separately by the CV-EAC.

**Death due to Other Cardiovascular Causes** refers to a cardiovascular death not included in the above categories [e.g. pulmonary embolism, cardiovascular intervention (other than one performed to treat an acute myocardial infarction or a complication of an acute myocardial infarction – see definition of death due to myocardial infarction, above), aortic aneurysm rupture, or peripheral arterial disease]. Mortal complications of cardiac surgery or non-surgical revascularization should be classified as cardiovascular deaths.

## 4.1.2 Non-cardiovascular deaths

A non-cardiovascular death is defined as any death that is not thought to be due to a cardiovascular cause. There should be unequivocal and documented evidence of a non-cardiovascular cause of death.

Further sub-classification of non-cardiovascular death will be as follows:

Pulmonary

Renal

Gastrointestinal

Infection (includes sepsis)

Non-infectious (e.g., systemic inflammatory response syndrome (SIRS))

Malignancy

Haemorrhage, not intracranial

Accidental/Trauma

Suicide

Non-cardiovascular surgery

Other non-cardiovascular, specify: ________________

## 4.1.3 Undetermined cause of death

This refers to any death not attributable to one of the above categories of cardiovascular death or to a non-cardiovascular cause (e.g. due to lack of information such as a case where the only information available is “patient died”). It is expected that every effort will be made to provide the adjudicating committee with enough information to attribute deaths to either a cardiovascular or non-cardiovascular cause so that the use of this category is kept to a minimal number of patients.

## 4.1.4 Non-fatal Cardiovascular Events

Date of onset

For purposes of classification, when classifying events that are a cause of hospitalization, the date of admission will be used as the onset date. In cases where the stated date of admission differs from the date the patient first presented to hospital with the event (e.g. because of a period of observation in an emergency department, medical assessment unit or equivalent), the date of initial presentation to hospital will be used (provided that the patient had not been discharged from hospital in the interim).

For events where an admission date is not applicable (or not available), the date of onset as stated by the investigator will be used.

## 4.2.1 Acute myocardial infarction

Note on biomarker elevations:

For cardiac biomarkers, laboratories should report an upper reference limit (URL). If the 99th percentile of the upper reference limit (URL) from the respective laboratory performing the assay is not available, then the URL for myocardial necrosis from the laboratory should be used. If the 99th percentile of the URL or the URL for myocardial necrosis is not available, the MI decision limit for the particular laboratory should be used as the URL.

## Spontaneous acute myocardial infarction:

A rise and/or fall of cardiac biomarkers (troponin or CK-MB) should usually be detected (see note below) with at least one value above the upper reference limit (URL) together with evidence of myocardial ischemia with at least one of the following:

- Clinical presentation consistent with ischemia
- ECG evidence of acute myocardial ischemia (as outlined in Table 1, below) or new left bundle branch block (LBBB).
- Development of pathological Q waves on the ECG (see Table 2, below)
- Imaging evidence of new loss of viable myocardium or new regional wall motion abnormality
- Autopsy evidence of acute myocardial infarction
- If biomarkers are elevated from a prior infarction, then a spontaneous myocardial infarction is defined as:

**a**. One of the following:

o Clinical presentation consistent with ischemia

o ECG evidence of acute myocardial ischemia (as outlined in Table 1, below) or new left bundle branch block. [The events committee will adjudicate in the context of the sequential ECG changes that are commonly seen in acute ST elevation/acute non-ST elevation myocardial infarction.]

o New pathological Q waves (see Table 2, below). [The events committee will adjudicate in the context of the sequential ECG changes that are commonly seen in acute ST elevation/acute non-ST elevation myocardial infarction.]

o Imaging evidence of new loss of viable myocardium or new regional wall motion abnormality

o Autopsy evidence of acute myocardial infarction

AND

**b**. Both of the following:

o Evidence that cardiac biomarker values were decreasing (e.g. two samples 3-6 hours apart) prior to the suspected acute myocardial infarction*

o ≥ 20% increase (and > URL) in troponin or CK-MB between a measurement made at the time of the initial presentation with the suspected recurrent myocardial infarction and a further sample taken 3-6 hours later

- *If biomarkers are increasing or peak is not reached, then a definite diagnosis of recurrent myocardial infarction is generally not possible.

Percutaneous coronary intervention-related acute myocardial infarction

Peri-percutaneous coronary intervention (PCI) acute myocardial infarction is defined by any of the following criteria. Symptoms of cardiac ischemia are not required.

Biomarker elevations within 48 hours of PCI:

• Troponin or CK-MB (preferred) > 5 x URL ***and***

• No evidence that cardiac biomarkers were elevated prior to the procedure;

OR

• Both of the following must be true:

o ≥ 50% increase in the cardiac biomarker result

o Evidence that cardiac biomarker values were decreasing (e.g. two samples 3-6 hours apart) prior to the suspected acute myocardial infarction

New pathological Q waves or new left bundle branch block (LBBB).

[If the PCI was undertaken in the context of an acute myocardial infarction, the events committee will adjudicate in the context of the sequential ECG changes that are commonly seen in acute ST elevation/acute non-ST elevation myocardial infarction.]

Autopsy evidence of acute myocardial infarction

Coronary artery bypass grafting-related acute myocardial infarction

Peri-coronary artery bypass graft surgery (CABG) acute myocardial infarction is defined by the following criteria. Symptoms of cardiac ischemia are not required.

Biomarker elevations within 72 hours of CABG:

• Troponin or CK-MB (preferred) > 10 x URL ***and***

• No evidence that cardiac biomarkers were elevated prior to the procedure;

OR

• Both of the following must be true:

o ≥ 50% increase in the cardiac biomarker result

o Evidence that cardiac biomarker values were decreasing (e.g. two samples 3-6 hours apart) prior to the suspected acute myocardial infarction

AND

One of the following:

New pathological Q-waves (preferably with evidence of persistence)

[If the CABG was undertaken in the context of an acute myocardial infarction, the events committee will adjudicate in the context of the sequential ECG changes that are commonly seen in acute ST elevation/acute non-ST elevation myocardial infarction.]

New LBBB (preferably with evidence of persistence)

Angiographically documented new graft or native coronary artery occlusion

Imaging evidence of new loss of viable myocardium

OR

Autopsy evidence of acute myocardial infarction

**Note:** For a diagnosis of acute myocardial infarction, a rise and/or fall of cardiac biomarkers should usually be detected. However, myocardial infarction may be adjudicated for an event that has characteristics which are very suggestive of acute infarction but which does not meet the strict definition because biomarkers are not available (e.g. not measured) or are non-contributory (e.g. may have normalized).

Suggestive characteristics are:

Typical cardiac ischemic-type pain/discomfort
(except for suspected acute myocardial infarction occurring in the context of PCI or CABG where this requirement need not apply)

AND

New ECG changes* or other evidence to support a diagnosis of acute myocardial infarction (e.g. imaging evidence of new loss of viable myocardium/new regional wall motion abnormality or angiography demonstrating occlusive coronary thrombus)

*If ECG tracings are not available for review, the adjudication may be made on the basis of reported ECG changes that have been clearly documented in the case records or in the case report form.

Clinical classification of different types of myocardial infarction

Myocardial infarctions will be clinically classified as:

Type 1

Spontaneous myocardial infarction related to ischemia due to a primary coronary event such as plaque erosion and/or rupture, fissuring, or dissection.

Type 2

Myocardial infarction secondary to ischemia due to either increased oxygen demand or decreased supply, e.g. coronary artery spasm, coronary embolism, anaemia, arrhythmias, hypertension, or hypotension.

Type 3

Sudden unexpected cardiac death, including cardiac arrest, often with symptoms suggestive of myocardial ischemia, accompanied by presumably new ST elevation, or new LBBB, or evidence of fresh thrombus in a coronary artery by angiography and/or at autopsy, but death occurring before blood samples could be obtained, or at a time before the appearance of cardiac biomarkers in the blood.

Type 4a

Myocardial infarction associated with PCI.

Type 4b

Myocardial infarction associated with stent thrombosis as documented by angiography or at autopsy.

Type 5

Myocardial infarction associated with CABG.

Myocardial infarctions will be further sub-classified as:

ST segment elevation myocardial infarction (STEMI).
**or**

Non-ST segment elevation myocardial infarction (NSTEMI).
**or**

Myocardial infarction, type (i.e. STEMI or NSTEMI) unknown.

| Table 1: ECG manifestations of acute myocardial ischemia (in absence of left ventricular hypertrophy and left bundle branch block) |
| --- |
| ST elevation  New ST elevation at the J-point in two anatomically contiguous leads with the cut-off  points: ≥ 0·2 mV in men (> 0·25 mV in men < 40 years) or ≥ 0·15 mV in women in leads V2-V3 and/or ≥ 0·1 mV in other leads.  ST depression and T wave changes  New horizontal or down-sloping ST depression ≥ 0·05 mV in two  contiguous leads; and/or new T wave inversion ≥ 0·1 mV in two contiguous  leads.  The above ECG criteria illustrate patterns consistent with myocardial ischemia. In patients with abnormal biomarkers, it is recognized that lesser ECG abnormalities may represent an ischemic response and may be accepted under the category of abnormal ECG findings. |

| Table 2: Pathological Q waves: |
| --- |
| Any Q-wave in leads V2-V3 ≥ 0·02 seconds or QS complex in leads V2 and V3  Q-wave ≥ 0·03 seconds and ≥ 0·1 mV deep or QS complex in leads I, II, aVL, aVF, or V4-V6 in any two leads of a contiguous lead grouping (I, aVL, V6; V4-V6; II, III, and aVF) a  A The same criteria are used for supplemental leads V7-V9, and for the Cabrera frontal plane lead grouping. |

## 4.2.2 Hospitalization for unstable angina

For the diagnosis of hospitalization due to unstable angina there should be emergency/unplanned admission to a hospital setting (emergency room, observation or inpatient unit) that results in at least one overnight stay (i.e. a date change) with fulfilment of the following criteria:

There should be:

1. Cardiac ischemic-type symptoms at rest (chest pain or equivalent) or an accelerating pattern of angina (e.g. exercise-related ischemic-type symptoms increasing in frequency and/or severity, decreasing threshold for onset of exercise related ischemic type symptoms) but without the fulfilment of the above diagnostic criteria for acute myocardial infarction.

and

2 The need for treatment with parenteral (intravenous, intra-arterial, buccal, transcutaneous or subcutaneous) anti-ischemic/antithrombotic therapy and/or coronary revascularization.

and

3a ECG manifestations of acute myocardial ischemia (New ST-T changes meeting the criteria for acute myocardial ischemia - as outlined in Table 1, section 5.2.1).

or

3b Angiographically significant coronary artery disease thought to be responsible for the patient’s presentation. [If both invasive and CT angiographic imaging of the coronary arteries were performed, the results of the invasive coronary angiogram should take preference.]

and

4 The independent clinician should be satisfied that unstable angina was the primary reason for hospitalization.

## 4.2.3 Hospitalization for other angina*

For the diagnosis of hospitalization for other angina, there should be emergency/unplanned admission to a hospital setting (emergency room, observation or inpatient unit) that results in at least one overnight stay (i.e. a date change) with fulfilment of the following criteria:

There should be:

Typical cardiac ischemic-type symptoms but without the fulfilment of the above diagnostic criteria for acute myocardial infarction or unstable angina.

and

2 The need for treatment with new or increased anti-anginal therapy (excluding sublingual nitrate therapy).

and

3a Investigations undertaken in view of the event (e.g. exercise ECG or stress myocardial perfusion scan) showing evidence of reversible myocardial ischemia.

or

3b Coronary angiography showing angiographically significant coronary disease thought to be responsible for the patient’s presentation. [If both invasive and CT angiographic imaging of the coronary arteries were performed, the results of the invasive coronary angiogram should take preference.]

and

4 The independent clinician should be satisfied that angina was the primary reason for hospitalization.

## 4.2.4 Hospitalization for other chest pain*

There should be:

Emergency/unplanned admission to a hospital setting (emergency room, observation or inpatient unit) that results in at least one overnight stay i.e. a date change) due to chest pain but where the definitions (above) of acute myocardial infarction, hospitalization for unstable angina or hospitalization for other angina are not met.

The independent clinician should be satisfied that chest pain was the primary reason for hospitalization.

*These events are not study cardiovascular events of interest but the definitions provided for these events will be used by the independent clinician to categorise reported myocardial infarction, angina and chest pain events that do not meet the study definition of acute myocardial infarction or hospitalization for unstable angina.

## 4.2.5 Stroke

**Stroke** is defined as an acute episode of neurological dysfunction caused by focal or global brain, spinal cord, or retinal vascular injury.

**A** For the diagnosis of stroke, the following 4 criteria should usually be fulfilled:

1. Rapid onset* of a focal/global neurological deficit with at least one of the following:

Change in level of consciousness

Hemiplegia

Hemiparesis

Numbness or sensory loss affecting one side of the body

Dysphasia/aphasia

Hemianopia (loss of half of the field of vision of one or both eyes)

Complete/partial loss of vision of one eye

Other new neurological sign(s)/symptom(s) consistent with stroke

*If the mode of onset is uncertain, a diagnosis of stroke may be made provided that there is no plausible non-stroke cause for the clinical presentation.

2. Duration of a focal/global neurological deficit > 24 hours

or

< 24 hours if

(i) this is because of at least one of the following therapeutic interventions:

(a) pharmacologic i.e. thrombolytic drug administration.

(b) non-pharmacologic i.e. neurointerventional procedure (e.g. intracranial angioplasty).

or

(ii) Brain imaging available clearly documenting a new haemorrhage or infarct.

or

(iii) the neurological deficit results in death

3. No other readily identifiable non-stroke cause for the clinical presentation (e.g. brain tumour, hypoglycaemia, peripheral lesion).

4. Confirmation of the diagnosis by at least one of the following**:

neurology or neurosurgical specialist.

brain imaging procedure (at least one of the following):

CT scan.

MRI scan.

cerebral vessel angiography.

Lumbar puncture (i.e. spinal fluid analysis diagnostic of intracranial haemorrhage).

B If the acute neurological deficit represents a worsening of a previous deficit, this worsened deficit must have:

Persisted for more than one week

**Or** < one week if

(i) this is because of at least one of the following therapeutic interventions:

(a) pharmacologic i.e. thrombolytic drug administration.

(b) non-pharmacologic i.e. neurointerventional procedure (e.g. intracranial angioplasty).

or

(ii) brain imaging available clearly documenting an appropriate new CT/MRI finding.

or

(iii) the neurological deficit results in death

Strokes will be further sub-classified as:

Ischemic (non-haemorrhagic) stroke

(i.e. caused by an infarction of central nervous system tissue)

or

Haemorrhagic stroke***

(i.e. caused by nontraumatic intraparenchymal, intraventricular or subarachnoid haemorrhage)

or

Stroke type (i.e. haemorrhagic or ischemic) unknown (i.e. when imaging/other investigations are unavailable or inconclusive).

***Subdural and extradural haemorrhages will be adjudicated (based on clinical signs and symptoms as well as neuroimaging and/or autopsy) and classified separately.

## 4.2.6. Heart Failure requiring hospitalization

For the diagnosis of heart failure requiring hospitalization, there should be emergency/unplanned admission to a hospital setting (emergency room, observation or inpatient unit) that results in at least one overnight stay (i.e. a date change) with fulfilment of the following criteria:

There should be:

Clinical manifestations of new or worsening heart failure including at least one of the following:

New or worsening dyspnoea on exertion

New or worsening dyspnoea at rest

New or worsening fatigue/decreased exercise tolerance

New or worsening orthopnoea

New or worsening PND (paroxysmal nocturnal dyspnoea)

New or worsening lower limb or sacral oedema

New or worsening pulmonary crackles/crepitations

New or worsening elevation of JVP (jugular venous pressure)

New or worsening third heart sound or gallop rhythm

**And**

1. Investigative evidence of structural or functional heart disease (if available) with at least *one* of the following:

Radiological evidence of pulmonary oedema/congestion or cardiomegaly.

Imaging ( e.g. echocardiography, cardiac magnetic resonance imaging, radionuclide ventriculography) evidence of an abnormality (e.g. left ventricular systolic dysfunction, significant valvular heart disease, left ventricular hypertrophy).

- - Elevation of BNP or NT-proBNP levels.
  - Other investigative evidence of structural or functional heart disease (e.g. evidence obtained from pulmonary artery catheterization).

**And**

**3** Need for new/increased therapy***** specifically for the treatment of heart failure

including at least one of the following:

New or increased oral therapy for the treatment of heart failure

(See note on oral therapy, below)

Initiation of intravenous diuretic, inotrope, vasodilator or other recognised intravenous heart failure treatment or up titration of such intravenous therapy if already receiving it

Mechanical or surgical intervention (e.g. mechanical or non-invasive ventilation, mechanical circulatory support, heart transplantation, ventricular pacing to improve cardiac function), or the use of ultrafiltration, hemofiltration, dialysis or other mechanical or surgical intervention that is specifically directed at treatment of heart failure.

Note on oral therapy: In general, for an event to qualify as *heart failure requiring hospitalization* on the basis of *oral* heart failure therapy (i.e. in cases where none of the non-pharmacological treatment modalities listed above have been utilized), the new or increased oral therapy should include oral diuretics. However, in special cases, other new or increased oral therapy (e.g. hydralazine/long acting nitrate, aldosterone antagonist) may be accepted provided that the adjudication committee is satisfied that:

the new or increased oral therapy was primarily directed at treating clinical manifestations of new or worsening heart failure (rather than, for example, initiation or up-titration of heart failure therapy as part of the routine optimization of medical therapy)

and

the totality of the evidence indicates that heart failure, rather than any other disease process, was the primary cause of the clinical presentation.

*****If time does not allow for the initiation of, or an increase in, treatment directed at heart failure or if the circumstances were such that doing so would have been inappropriate (e.g. patient refusal), the independent clinician will adjudicate on clinical presentation and, if available, investigative evidence.

and

**4** The independent clinician should be satisfied that heart failure was the primary disease process accounting for the clinical presentation.

## 4.2.7. Renal Failure requiring hospitalization

Contrast-induced nephropathy: is defined as either a greater than 25% increase of serum creatinine or an absolute increase in serum creatinine of 0·5 mg/dL after a radiographic examination using a contrast agent.

## 4.2.8. Bleeding requiring hospitalization

Bleeding: is defined according to the ACUITY criteria: major bleed = intracranial or intraocular bleeding; bleeding at the site of angiography requiring intervention; a hematoma of 5 cm in diameter; a reduction in haemoglobin level of at least 4 g/dL in the absence of overt bleeding or 3 g/dL with a source of bleeding; or transfusion.

## 6.1 Event identification

The BHF MR-MI study will use paper-based and electronic data capture (EDC). Those events requiring independent validation (see section 4) will be reported by the Investigator via the EDC (electronic data capture) system.

## 6.2 Incomplete event data

If, having reviewed the event data pertaining to an event, the independent cardiologist deems that the information provided is insufficient for the purposes of event adjudication, an electronic request for further information detailing the information required will be made. The date of request will be recorded electronically and the event will be classified as not adjudicated/pending additional information.

# Clinical data to be provided

The trial management team (including Prof Berry, Dr Carrick, Ms Joanne Kelly CRN) will provide event data for each potential cardiovascular event requiring adjudication to the independent cardiologist.

Data to be included for event classification will include:

Subject study identification number and event details

On request: Relevant de-identified CRF data (including any relevant event-specific CRFs e.g. the *myocardial infarction/hospitalization for unstable angina/other angina/*

*chest pain* event form).

Supportive source documentation as required

Baseline and subsequent scheduled ECGs obtained during study participation.

All clinical data would be de-identified.

De-identified Source Documentation

The following source documents (if available) will be provided to the independent cardiologist as part of the standard dossier contents for cardiovascular events requiring review/adjudication:

Death

Hospital Discharge Summary/Death Summary

Autopsy Report

Death Certificate

Admission History & Physical (if applicable)

Acute Myocardial Infarction/Hospitalization for Unstable Angina/Other Angina/Chest Pain

Hospital Discharge Summary

ECGs

Pre-Randomization/Screening

Baseline (prior to event but post-randomization)

During Event

Post-Event

Relevant Procedure/Operation Reports

Relevant Laboratory Reports (e.g. that document the cardiac enzyme/marker measurements provided – peak values and pre-procedure and post-procedure values, where applicable)

Reports for other investigations taken:

PCI Report

CABG Report

Coronary Angiography Report

Echocardiogram Report

Exercise ECG Report

Stress Myocardial Perfusion Scan Report

Other investigation report undertaken to test for presence of reversible myocardial ischemia

Admission History & Physical

Stroke/TIA/Other cerebrovascular events

Hospital Discharge Summary

Neurology Consultation Report(s)

Reports for other investigations undertaken:

CT Brain Scan Report

MRI Brain Scan Report

Cerebral Angiography Report

Lumbar Puncture Report

Admission History & Physical

Heart Failure requiring hospitalization

Hospital Discharge Summary

Chest X-Ray Report

Prescription Sheets/Medication Administration Records

Echocardiogram Report

Relevant Laboratory Reports (e.g. for peak BNP/NT-proBNP)

Reports for other investigations undertaken:

Cardiac Magnetic Resonance Imaging

Radionuclide Ventriculogram Scan

Pulmonary Artery Catherization

Admission History & Physical

Coronary revascularization procedure

Hospital Discharge Summary

Relevant Procedure/Operation Reports

Bleeding

Hospital Discharge Summary

Relevant Procedure/Operation Reports

Hb

Blood transfusion results

Diagnostic and therapeutic procedures (e.g. gastroscopy).

## References

1. Kramer CM, Barkhausen J, Flamm SD, Kim RJ, Nagel E. Standardized cardiovascular magnetic resonance (CMR) protocols 2013 update. Journal of cardiovascular magnetic resonance : official journal of the Society for Cardiovascular Magnetic Resonance 2013;**15**:91.

2. Moon JC, Messroghli DR, Kellman P, Piechnik SK, Robson MD, Ugander M, Gatehouse PD, Arai AE, Friedrich MG, Neubauer S, Schulz-Menger J, Schelbert EB, Society for Cardiovascular Magnetic Resonance I, Cardiovascular Magnetic Resonance Working Group of the European Society of C. Myocardial T1 mapping and extracellular volume quantification: a Society for Cardiovascular Magnetic Resonance (SCMR) and CMR Working Group of the European Society of Cardiology consensus statement. Journal of cardiovascular magnetic resonance : official journal of the Society for Cardiovascular Magnetic Resonance 2013;**15**:92.

3. Cerqueira MD, Weissman NJ, Dilsizian V, Jacobs AK, Kaul S, Laskey WK, Pennell DJ, Rumberger JA, Ryan T, Verani MS. Standardized myocardial segmentation and nomenclature for tomographic imaging of the heart. A statement for healthcare professionals from the Cardiac Imaging Committee of the Council on Clinical Cardiology of the American Heart Association. Circulation 2002;**105**(4):539-42.

4. Steg PG, James SK, Atar D, Badano LP, Blomstrom-Lundqvist C, Borger MA, Di Mario C, Dickstein K, Ducrocq G, Fernandez-Aviles F, Gershlick AH, Giannuzzi P, Halvorsen S, Huber K, Juni P, Kastrati A, Knuuti J, Lenzen MJ, Mahaffey KW, Valgimigli M, van 't Hof A, Widimsky P, Zahger D. ESC Guidelines for the management of acute myocardial infarction in patients presenting with ST-segment elevation. European heart journal 2012;**33**(20):2569-619.

5. Windecker S, Kolh P, Alfonso F, Collet JP, Cremer J, Falk V, Filippatos G, Hamm C, Head SJ, Juni P, Kappetein AP, Kastrati A, Knuuti J, Landmesser U, Laufer G, Neumann FJ, Richter DJ, Schauerte P, Sousa Uva M, Stefanini GG, Taggart DP, Torracca L, Valgimigli M, Wijns W, Witkowski A. 2014 ESC/EACTS Guidelines on myocardial revascularization: The Task Force on Myocardial Revascularization of the European Society of Cardiology (ESC) and the European Association for Cardio-Thoracic Surgery (EACTS)Developed with the special contribution of the European Association of Percutaneous Cardiovascular Interventions (EAPCI). European heart journal 2014;**35**(37):2541-619.

6. Messroghli DR, Radjenovic A, Kozerke S, Higgins DM, Sivananthan MU, Ridgway JP. Modified Look-Locker inversion recovery (MOLLI) for high-resolution T1 mapping of the heart. Magnetic resonance in medicine : official journal of the Society of Magnetic Resonance in Medicine / Society of Magnetic Resonance in Medicine 2004;**52**(1):141-6.

7. Messroghli DR, Walters K, Plein S, Sparrow P, Friedrich MG, Ridgway JP, Sivananthan MU. Myocardial T1 mapping: application to patients with acute and chronic myocardial infarction. Magnetic resonance in medicine : official journal of the Society of Magnetic Resonance in Medicine / Society of Magnetic Resonance in Medicine 2007;**58**(1):34-40.

8. Giri S, Chung YC, Merchant A, Mihai G, Rajagopalan S, Raman SV, Simonetti OP. T2 quantification for improved detection of myocardial oedema. Journal of cardiovascular magnetic resonance : official journal of the Society for Cardiovascular Magnetic Resonance 2009;**11**:56.

9. Verhaert D, Thavendiranathan P, Giri S, Mihai G, Rajagopalan S, Simonetti OP, Raman SV. Direct T2 quantification of myocardial oedema in acute ischemic injury. JACC Cardiovascular imaging 2011;**4**(3):269-78.

10. Kellman P, Arai AE, McVeigh ER, Aletras AH. Phase-sensitive inversion recovery for detecting myocardial infarction using gadolinium-delayed hyperenhancement. Magnetic resonance in medicine : official journal of the Society of Magnetic Resonance in Medicine / Society of Magnetic Resonance in Medicine 2002;**47**(2):372-83.

11. Xue H, Greiser A, Zuehlsdorff S, Jolly MP, Guehring J, Arai AE, Kellman P. Phase-sensitive inversion recovery for myocardial T1 mapping with motion correction and parametric fitting. Magnetic resonance in medicine : official journal of the Society of Magnetic Resonance in Medicine / Society of Magnetic Resonance in Medicine 2013;**69**(5):1408-20.

12. Flett AS, Hasleton J, Cook C, Hausenloy D, Quarta G, Ariti C, Muthurangu V, Moon JC. Evaluation of techniques for the quantification of myocardial scar of differing etiology using cardiac magnetic resonance. JACC Cardiovascular imaging 2011;**4**(2):150-6.

13. Wassmuth R, Prothmann M, Utz W, Dieringer M, von Knobelsdorff-Brenkenhoff F, Greiser A, Schulz-Menger J. Variability and homogeneity of cardiovascular magnetic resonance myocardial T2-mapping in volunteers compared to patients with oedema. Journal of cardiovascular magnetic resonance : official journal of the Society for Cardiovascular Magnetic Resonance 2013;**15**:27.

14. Eitel I, Desch S, Fuernau G, Hildebrand L, Gutberlet M, Schuler G, Thiele H. Prognostic significance and determinants of myocardial salvage assessed by cardiovascular magnetic resonance in acute reperfused myocardial infarction. Journal of the American College of Cardiology 2010;**55**(22):2470-9.

15. Berry C, Kellman P, Mancini C, Chen MY, Bandettini WP, Lowrey T, Hsu LY, Aletras AH, Arai AE. Magnetic resonance imaging delineates the ischemic area at risk and myocardial salvage in patients with acute myocardial infarction. Circulation Cardiovascular imaging 2010;**3**(5):527-35.

16. Payne AR, Casey M, McClure J, McGeoch R, Murphy A, Woodward R, Saul A, Bi X, Zuehlsdorff S, Oldroyd KG, Tzemos N, Berry C. Bright-blood T2-weighted MRI has higher diagnostic accuracy than dark-blood short tau inversion recovery MRI for detection of acute myocardial infarction and for assessment of the ischemic area at risk and myocardial salvage. Circulation Cardiovascular imaging 2011;**4**(3):210-9.

17. Francone M, Bucciarelli-Ducci C, Carbone I, Canali E, Scardala R, Calabrese FA, Sardella G, Mancone M, Catalano C, Fedele F, Passariello R, Bogaert J, Agati L. Impact of primary coronary angioplasty delay on myocardial salvage, infarct size, and microvascular damage in patients with ST-segment elevation myocardial infarction: insight from cardiovascular magnetic resonance. Journal of the American College of Cardiology 2009;**54**(23):2145-53.

18. Payne AR, Berry C, Doolin O, McEntegart M, Petrie MC, Lindsay MM, Hood S, Carrick D, Tzemos N, Weale P, McComb C, Foster J, Ford I, Oldroyd KG. Microvascular Resistance Predicts Myocardial Salvage and Infarct Characteristics in ST-Elevation Myocardial Infarction. Journal of the American Heart Association 2012;**1**(4):e002246.

19. van Kranenburg M, Magro M, Thiele H, de Waha S, Eitel I, Cochet A, Cottin Y, Atar D, Buser P, Wu E, Lee D, Bodi V, Klug G, Metzler B, Delewi R, Bernhardt P, Rottbauer W, Boersma E, Zijlstra F, van Geuns RJ. Prognostic value of microvascular obstruction and infarct size, as measured by CMR in STEMI patients. JACC Cardiovascular imaging 2014;**7**(9):930-9.

20. Pencina MJ, D'Agostino RB, Sr., Steyerberg EW. Extensions of net reclassification improvement calculations to measure usefulness of new biomarkers. Statistics in medicine 2011;**30**(1):11-21.

21. Thygesen K, Alpert JS, Jaffe AS, Simoons ML, Chaitman BR, White HD. Third universal definition of myocardial infarction. Global heart 2012;**7**(4):275-95.

22. Karen A. Hicks HMJH, Kenneth W. Mahaffey, Roxana Mehran, Steven E. Nissen, Norman L. Stockbridge, Shari L. Targum, Robert Temple. *on behalf of the Standardized Data Collection for Cardiovascular Trials Initiative Standardized Definitions for End Point Events in Cardiovascular Trials.*[*http://www.cdisc.org/stuff/contentmgr/files/0/2356ae38ac190ab8ca4ae0b222392b37/misc/cdisc_november_16__2010.pdf*](http://www.cdisc.org/stuff/contentmgr/files/0/2356ae38ac190ab8ca4ae0b222392b37/misc/cdisc_november_16__2010.pdf) <http://www.cdisc.org/stuff/contentmgr/files/0/2356ae38ac190ab8ca4ae0b222392b37/misc/cdisc_november_16__2010.pdf>.
