# Supplementary material for: Prognostic significance of infarct core pathology revealed by quantitative non-contrast in comparison with contrast cardiac magnetic resonance imaging in reperfused ST-elevation myocardial infarction survivors
Source: Eur Heart J. 2015 Aug 10;37(13):1044–59. doi: 10.1093/eurheartj/ehv372 (PMC4816961; doi:10.1093/eurheartj/ehv372)
Supplement: Supplementary Data [file ehv372_supplementary_data.zip › ehv372supp_data2.docx]

# Supplementary Results

The intra-class correlation coefficient (ICC) for the reliability of infarct core native T1 values measured independently by 2 observers in 12 randomly selected STEMI patients was 0.91 (0.71, 0.97) (p<0.001).

Fifty healthy volunteers from the same geographical region (52% male, mean (SD) age 54 (13) years) without a history of cardiovascular disease or therapy and who had a normal electrocardiogram were enrolled during the same time period as the STEMI patients. These individuals volunteered based on advertising within the public areas of the hospital and the University of Glasgow, and through personal contacts of the researchers. The volunteers were scanned using the same 1.5 Tesla MRI scanner (Siemens AVANTO) as the STEMI patients, and the approach to image analysis was the same as for STEMI patients also, including regional segmentation of the left ventricle according to the American Heart Association model [28].

In healthy subjects, mid-ventricular T1 values were lower in males than in females (948 (20) ms vs. 968 (23) ms; p=0.003). In both men and women, the infero-lateral segment had the highest T1 compared to the antero-septal segment (960 (28) ms vs. 939 (26) ms and 978 (32) ms vs. 961 (34) ms, respectively; p<0.001 and p=0.011). At the mid-ventricular level, mean remote zone native T1 was similar in STEMI patients (961 (25) ms) and healthy volunteers (958 (24); p=0.314).

The coefficients of variation (CoV) for native T1 in the mid-ventricular level with regions-of-interest within myocardial regions were: anterior segment CoV = 2.35; antero-lateral segment CoV = 2.98; antero-septal segment CoV = 3.35; inferior segment CoV = 2.49; infero-lateral segment CoV = 3.22; infero-septal segment CoV = 2.90.

## Univariable associates of native T1 values in the infarct core early post-MI

160 STEMI survivors had evidence of infarct core pathology revealed by native T1 mapping with CMR 2 days post-MI. The clinical characteristics that were univariably associated with infarct core native T1 time (ms) in these subjects and were included in the multivariable models were systolic blood pressure at initial angiography, mmHg (-0.45 (-0.85, -0.05); p = 0.026), LV ejection fraction (%) (0.94 (-0.03, 1.91); p=0.057), infarct size (% LV mass) (-0.93 (-1.68, -0.18); p=0.016), minimum leucocyte count (x10^9^L) (-3.97 (-6.93, -1.01); p=0.009), minimum neutrophil count (x10^9^L) (-4.99 (-8.41, -1.56); p=0.005), maximum log CRP (-7.35 (-15.00, 0.31); p=0.060), maximum leucocyte count (x10^9^L) (-2.53 (-5.09, 0.02); p=0.052), and maximum monocyte count (x10^9^L) (-21.83 (-42.15, -1.51); p=0.035).

## Univariable associates of adverse LV remodelling revealed by CMR in STEMI survivors after 6 months follow-up

There were 20 clinical characteristics that were univariable associates of adverse LV remodeling, defined as an increase in LV end-diastolic volume ≥ 20% at 6 months from baseline [3]. 244 STEMI participants had complete data for these clinical characteristics and paired CMR scans at baseline and follow-up, and 136 of these patients had a hypointense native T1 core. The univariable characteristics and their p-values that were included in the multivariable model were: **infarct core native T1 (p=0.052),** age (p=0.909), male sex (p=0.847), body mass index (p=0.366), previous myocardial infarction (p=0.364), diabetes mellitus (p=0.491), previous percutaneous coronary intervention (p=0.639), **cigarette smoking (p=0.036),** history of hypertension (p=0.463), hypercholesterolaema (p=0.912), history of angina (p=0.972), heart rate (p=0.569), systolic blood pressure at initial angiography (p=0.718), Killip class II vs. Killip class I (reference category) (p=0.437), Killip class III/IV vs. Killip class I (reference category) (p=0.574), **sustained ventricular arrhythmia (p=0.015),** symptom onset to reperfusion time (p=0.793), TIMI flow grade 2/3 vs. grade 1 (reference category) at initial angiography (p=0.648), ST segment resolution (none vs. complete (reference category), and p=0.966; incomplete vs. complete (reference category), p=0.089).

## Linear vs. non-linear relationships between infarct core T1 and LV outcomes

Loess plots were used to examine the linearity of relationships between T1 core and each of LVEF and LVEDV at baseline and follow-up.


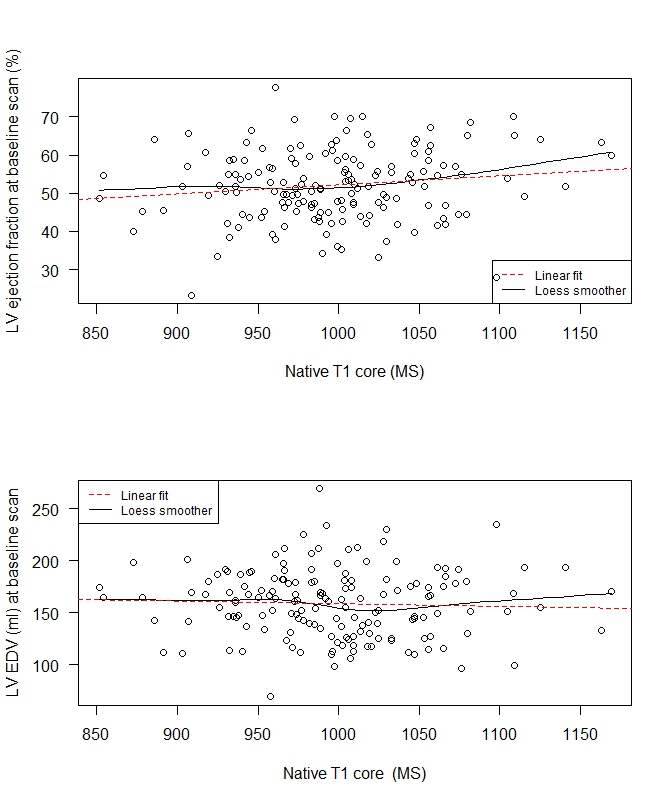


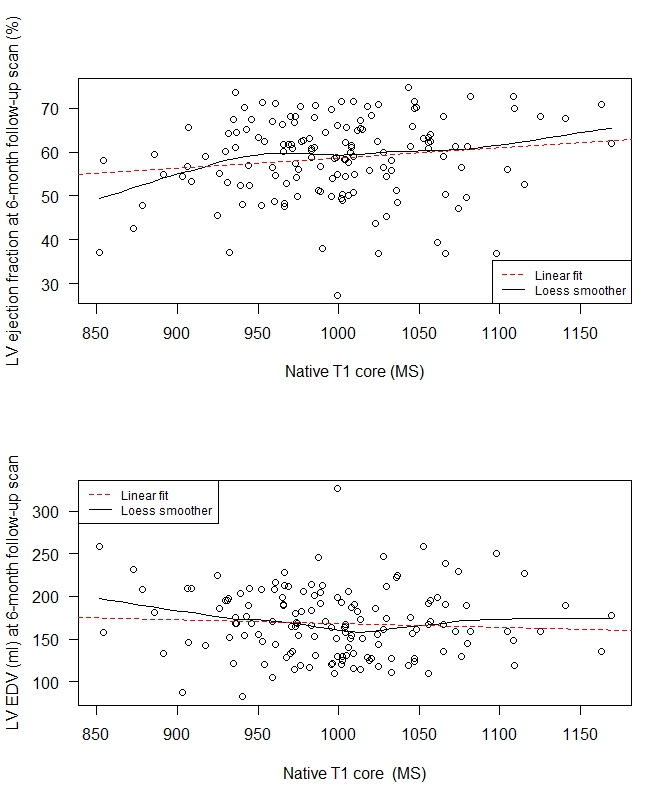


From examining the Loess plots, the relationships between T1 core and LV parameters were reasonably linear. In other words, there was no evidence of non-linearity between infarct core T1 (ms) and LV outcomes.

## Infarct core native T1 early post-MI and NT-proBNP, a biochemical measure of left ventricular remodelling, at 6 months

Biomarker blood samples were collected in the STEMI patients who had been enrolled during office hours and NT-proBNP results were available in 151 (52%) of 288 STEMI patients overall. 81 of these STEMI patients had evaluable T1 CMR maps at baseline and an NT-proBNP result at 6 months, and 50 (62%) of these patients had a hypointense core disclosed by T1 mapping. The characteristics of these patients were similar to those of the whole cohort (Supplementary Tables 4 and 5).

Native T1 within the infarct core zone and NT-proBNP were not associated at baseline (Table 1). T1 values within the infarct core at baseline were associated with log NT-proBNP (per 1 pg/mL change) at 6 months (per 1 ms reduction in native T1: coefficient (95% CI) 0.01 (0.01, 0.00); p=0.015) (n=50), independent of LV end-diastolic volume and NT-proBNP at baseline.

**Supplementary Table 1.** The results from the initial blood test taken on admission to hospital, the peak value obtained from subsequent blood tests during the first 48 hours in-hospital, and the maximum change from the admission result.

| Blood results |  | All STEMI patients  n=288 |
| --- | --- | --- |
| *Initial result on admission* |  |  |
| C-reactive protein, (mg/L) | median (IQR)  range, g/L | 3.0 (2.0 - 7.0)  0 - 265.0 |
| Leucocyte cell count (x10^9^L) |  | 12.36 (3.48) |
| Neutrophil count (x10^9^L) |  | 9.56 (3.24) |
| Monocytes (x10^9^L) |  | 0.84 (0.36) |
| Eosinophils (x10^9^L) |  | 0.12 (0.13) |
| Lymphocytes (x10^9^L) |  | 1.79 (0.79) |
| Basophils (x10^9^L) |  | 0.03 (0.02) |
| *Maximum change within 2 days from admission* |  |  |
| C-reactive protein, (mg/L) | median (IQR)  range, g/L | 3.0 (0.0 - 9.0)  0 - 266.0 |
| Leucocyte cell count (x10^9^L) |  | 2.53 (2.02) |
| Neutrophil count (x10^9^L) |  | 2.87 (2.14) |
| Monocytes (x10^9^L) |  | 0.31 (0.25) |
| Eosinophils (x10^9^L) |  | 0.08 (0.08) |
| Lymphocytes (x10^9^L) |  | 0.72 (0.53) |
| Basophils (x10^9^L) |  | 0.01 (0.01) |

Reference range in SI units: C-reactive protein (mg/L) < 10; leucocyte count (x10^9^/L) 4.0-11.0 (x10^9^/L); neutrophil count (x10^9^/L) 2.0 –7.5; lymphocyte count (x10^9^/L) 1.50 – 4.0; monocytes (x10^9^/L) 0.2 - 0.80; eosinophils (x10^9^/L) 0.04 - 0.40; basophils (x10^9^/L) 0.02 - 0.10.

**Supplementary Table 2.**

|  | Native T1 infarct core absent | Native T1 infarct core present |  |
| --- | --- | --- | --- |
| T2 core |  |  |  |
| T2 core absent | 111 | 2 | Specificity 98.2 %  95% CI (95.8, 100.0) |
| T2 core present | 17 | 158 | Sensitivity 90.3%  95% CI (85.9, 94.7) |
|  | NPV 86.7%  95% CI (80.8, 92.9) | PPV 98.8%  95% CI (97.0, 100.0) |  |
| Myocardial haemorrhage |  |  |  |
| Myocardial haemorrhage absent | 97 | 47 | Specificity 67.4% 95% CI (60.5, 74.8) |
| Myocardial haemorrhage present | 2 | 94 | Sensitivity 97.9% 95% CI (95.2, 100.0) |
|  | NPV 97.9%  95% CI (95.3, 100.0) | PPV 66.7%  95% CI (59.4, 74.2) |  |
| Early MVO |  |  |  |
| Early MVO absent | 114 | 10 | Specificity 91.9% 95% CI (87.1, 97.1) |
| Early MVO present | 14 | 150 | Sensitivity 91.5% 95% CI (87.3, 95.7) |
|  | NPV 89.1%  95% CI (83.8, 94.6) | PPV 93.8%  95% CI (90.1, 97.7) |  |
| Late MVO |  |  |  |
| Late MVO absent | 120 | 23 | Specificity 83.9% 95% CI (78.4, 89.7) |
| Late MVO present | 8 | 137 | Sensitivity 94.5% 95% CI (90.8, 98.3) |
|  | NPV 93.8%  95% CI (89.6, 98.1) | PPV 85.6%  95% CI (0.80, 0.91) |  |

95% CI – 95% confidence interval; MVO – microvascular obstruction; NPV – negative predictive value; PPV – positive predictive value. The CMR approaches for delineation of early MVO, late MVO and myocardial haemorrhage are described in the Methods.

**Supplementary Table 3.** Clinical and angiographic characteristics of 81 STEMI patients who had CMR with evaluable T1 maps at baseline and NT-proBNP results at 6 months. The patients are grouped according to tertiles of hypointense core T1 values (ms) at baseline.

| Characteristics* |  | All patients | Patients with a native T1 infarct core grouped by tertile of infarct core zone native T1 (ms) at baseline | | |  |
| --- | --- | --- | --- | --- | --- | --- |
|  |  |  | ≤ 973 ms | > 973 to ≤ 1014 ms | > 1014 ms | P-value |
|  |  | n=81 | n = 30 | n = 29 | n = 22 |  |
| Age, years |  | 59 (11) | 58 (11) | 59 (12) | 59 (11) | 0.925 |
| Male sex, n (%) |  | 67 (83) | 26 (87) | 22 (76) | 19 (86) | 0.570 |
| BMI, (kg/m^2^) |  | 28 (4) | 30 (4) | 28 (4) | 28 (5) | 0.352 |
| Hypertension, n (%) |  | 25 (31) | 7 (23) | 13 (45) | 5 (23) | 0.146 |
| Current smoking, n (%) |  | 45 (56) | 19 (63) | 15 (52) | 11 (50) | 0.587 |
| Hypercholesterolemia, n (%) |  | 19 (24) | 6 (20) | 7 (24) | 6 (27) | 0.849 |
| Diabetes mellitus‡, n (%) |  | 6 (7) | 1 (3) | 3 (10) | 2 (9) | 0.571 |
| Previous angina, n (%) |  | 9 (11) | 4 (13) | 3 (10) | 2 (9) | 1.000 |
| Previous myocardial infarction, n (%) |  | 8 (10) | 2 (7) | 2 (7) | 4 (18) | 0.450 |
| Previous PCI, n (%) |  | 7 (9) | 1 (3) | 1 (3) | 5 (23) | 0.031 |
| *Presenting characteristics* |  |  |  |  |  |  |
| Heart rate, bpm |  | 78 (15) | 82 (15) | 78 (15) | 72 (15) | 0.059 |
| Systolic blood pressure, mmHg |  | 138 (22) | 140 (24) | 141 (22) | 133 (19) | 0.419 |
| Diastolic blood pressure, mmHg |  | 81 (14) | 84 (16) | 82 (13) | 75 (13) | 0.071 |
| Time from symptom onset to reperfusion, min |  | 171 (116, 254) | 219 (140, 298) | 134 (110, 213) | 208 (125, 257) | 0.433 |
| Ventricular fibrillation†, n (%) |  | 4 (5) | 1 (3) | 1 (3) | 2 (9) | 0.672 |
| Heart failure Killip class, n (%) | I | 54 (67) | 14 (47) | 24 (83) | 16 (73) |  |
|  | II | 21 (26) | 11 (37) | 4 (14) | 6 (27) | 0.019 |
|  | III or IV | 6 (7) | 5 (17) | 1 (3) | 0 (0) |  |
| ECG |  |  |  |  |  |  |
| ST segment elevation resolution post PCI, n (%) |  |  |  |  |  |  |
| Complete, ≥70 % |  | 29 (36) | 10 (33) | 13 (45) | 6 (27) |  |
| Incomplete, 30% to < 70% |  | 35 (43) | 15 (50) | 10 (35) | 10 (46) | 0.621 |
| None, ≤30% |  | 17 (21) | 5 (17) | 6 (21) | 6 (27) |  |
| *Coronary angiography* |  |  |  |  |  |  |
| Reperfusion strategy, n (%) |  |  |  |  |  |  |
| Primary PCI |  | 77 (95) | 28 (93) | 27 (93) | 22 (100) |  |
| Rescue PCI (failed thrombolysis) |  | 35 (43) | 2 (7) | 2 (7) | 0 (0) | 0.551 |
| Successful thrombolysis |  | 29 (36) | 0 (0) | 0 (0) | 0 (0) |  |
| Number of diseased arteries¥, n (%) | 1 | 37 (46) | 11 (37) | 12 (41) | 14 (64) |  |
|  | 2 | 26 (32) | 11 (37) | 10 (35) | 5 (23) | 0.246 |
|  | 3 | 17 (21) | 8 (27) | 7 (24) | 2 (9) |  |
|  | LM | 1 (1) | 0 (0) | 0 (0) | 1 (%) |  |
| Culprit artery, n (%) | LAD | 31 (38) | 11 (37) | 11 (38) | 9 (41) |  |
|  | LCX | 14 (17) | 4 (13) | 7 (24) | 3 (14) | 0.806 |
|  | RCA | 36 (44) | 15 (50) | 11 (38) | 10 (46) |  |
| TIMI coronary flow grade pre-PCI, n (%) | 0/1 | 65 (80) | 27 (90) | 21 (72) | 17 (77) |  |
|  | 2 | 13 (16) | 3 (10) | 6 (21) | 4 (18) | 0.202 |
|  | 3 | 3 (4) | 0 (0) | 2 (7) | 1 (5) |  |
| TIMI coronary flow grade post-PCI, n (%) | 0/1 | 0 (0) | 0 (0) | 0 (0) | 0 (0) |  |
|  | 2 | 1 (1) | 1 (3) | 0 (0) | 0 (0) | - |
|  | 3 | 80 (99) | 29 (97) | 29 (100) | 22 (100) |  |
| Medical therapy |  |  |  |  |  |  |
| ACE-I or ARB |  | 81 (100) | 30 (100) | 29 (100) | 22 (100) | - |
| Beta-blocker |  | 80 (99) | 30 (100) | 28 (97) | 22 (100) | 0.630 |
| *Initial blood results on admission* |  |  |  |  |  |  |
| C-reactive protein, (mg/L) | Median (IQR)  Range | 3.0 (2.0, 7.5)  1.0, 68.0 | 3.0 (2.0, 9.5)  1.0, 35.0 | 3.0 (1.0, 5.0)  1.0, 24.0 | 4.0 (1.3, 7.8)  1.0, 68.0 | 0.261 |
| Leucocyte cell count (x10^9^L) |  | 12.4 (3.3) | 12.5 (3.1) | 12.9 (3.5) | 11.7 (3.4) | 0.447 |
| Neutrophil count (x10^9^L) |  | 9.8 (3.1) | 9.5 (3.0) | 10.4 (3.2) | 9.2 (2.9) | 0.309 |
| Monocytes (x10^9^L) |  | 0.9 (0.3) | 1.0 (0.3) | 0.8 (0.3) | 0.8 (0.4) | 0.162 |

Footnote: ACE-I or ARB = angiotensin converting enzyme inhibitor or angiotensin receptor blocker; LAD = Left anterior descending coronary artery; LCX = Left circumflex coronary artery; LM = left main coronary artery; RCA = right coronary artery; PCI = percutaneous coronary intervention; TIMI = Thrombolysis in Myocardial Infarction grade. Killip classification of heart failure after acute myocardial infarction: class I - no heart failure, class II - pulmonary rales or crepitations, a third heart sound, and elevated jugular venous pressure, class III - acute pulmonary edema, class IV - cardiogenic shock. * Data are given as n (%) or mean (SD). ‡ Diabetes mellitus was defined as a history of diet-controlled or treated diabetes. † Successfully electrically cardioverted ventricular fibrillation at presentation or during emergency PCI procedure. ¥ Multivessel coronary artery disease was defined according to the number of stenoses of at least 50% of the reference vessel diameter, by visual assessment and whether or not there was left main stem involvement. Data are reported as mean (SD), median (IQR), or N (%) as appropriate. P-values have been obtained from a one-way ANOVA or Fisher test. TIMI flow grades pre- and post-PCI were grouped 0/1 vs. 2/3 for this analysis.

**Supplementary Table 4.** CMR observations in 81 STEMI patients who had evaluable T1 maps at baseline and NT-proBNP results at 6 months. The patients are grouped according to tertiles of hypointense core T1 values (ms) at baseline.

| Characteristics* | All patients | Patients with a native T1 infarct core grouped by tertile of infarct core zone native T1 (ms) at baseline | | | P-value |
| --- | --- | --- | --- | --- | --- |
|  |  | ≤ 973 ms | > 973 to ≤ 1014 ms | > 1014 ms |  |
|  | n = 81 | n = 30 | n = 29 | n = 22 |  |
| *CMR findings 2 days post-MI* |  |  |  |  |  |
| LV ejection fraction, % | 52 (9) | 52 (10) | 51 (8) | 55 (9) | 0.349 |
| LV end-diastolic volume, ml |  |  |  |  |  |
| Men | 165 (26) | 165 (20) | 163 (32) | 168 (27) | 0.814 |
| Women | 128 (20) | 137 (28) | 129 (17) | 112 (12) | 0.304 |
| LV end-systolic volume, ml |  |  |  |  |  |
| Men | 79 (67, 94) | 74 (67, 90) | 81 (73, 93) | 79 (63, 100) | 0.878 |
| Women | 61 (47, 66) | 62 (57, 73) | 65 (53, 67) | 45 (40, 49) | 0.141 |
| LV mass, g |  |  |  |  |  |
| Men | 148 (129, 168) | 149 (135, 171) | 142 (117, 154) | 151 (130, 171) | 0.339 |
| Women | 102 (90, 133) | 116 (92, 140) | 126 (101, 133) | 83 (75, 90) | 0.150 |
| *Edema and infarct characteristics* |  |  |  |  |  |
| Area at risk, % LV mass | 35 (11) | 36 (11) | 35 (11) | 35 (12) | 0.853 |
| Infarct size, % LV mass | 22 (14, 31) | 25 (15, 32) | 25 (14, 31) | 19 (14, 29) | 0.444 |
| Myocardial salvage, % of LV mass | 17 (11, 24) | 17 (14, 24) | 16 (10, 23) | 18 (11, 28) | 0.483 |
| Myocardial salvage index, % of LV mass | 51 (41, 64) | 51 (42, 62) | 47 (32, 64) | 56 (43, 67) | 0.527 |
| Late microvascular obstruction present, n (%) | 66 (82) | 26 (87) | 23 (79) | 17 (77) | 0.633 |
| Late microvascular obstruction, % LV mass | 2.7 (0.7, 7.5) | 4.9 (1.7, 10.7) | 2.5 (0.7, 7.5) | 0.9 (0.3, 3.2) | 0.032 |
| *Myocardial native T1 values* |  |  |  |  |  |
| T1 remote myocardium (all subjects), ms | 963 (24) | 961 (27) | 961 (16) | 968 (28) | 0.489 |
| Men, ms | 963 (24) | 961 (27) | 961 (18) | 968 (27) | 0.565 |
| Women, ms | 964 (21) | 961 (27) | 963 (10) | 970 (38) | 0.864 |
| T1 infarct zone, ms | 1087 (47) | 1053 (38) | 1087 (30) | 1132 (41) | <0.001 |
|  |  |  |  |  |  |
| T1 hypointense infarct core, ms | 990 (53) | 939 (30) | 994 (12) | 1055 (34) | <0.001 |
| *CMR findings 6 months post-MI* |  |  |  |  |  |
| LV ejection fraction at 6 months, % | 61 (54, 66) | 59 (53, 64) | 61 (54, 64) | 62 (56, 68) | 0.368 |
| LV end-diastolic volume at 6 months, ml |  |  |  |  |  |
| Men | 174 (156, 206) | 186 (155, 209) | 171 (156, 197) | 167 (158, 191) | 0.677 |
| Women | 123 (113, 132) | 120 (100, 137) | 129 (123, 131) | 111 (110, 115) | 0.180 |
| LV end-systolic volume at 6 months, ml |  |  |  |  |  |
| Men | 70 (57, 90) | 74 (57, 98) | 71 (61, 84) | 63 (53, 95) | 0.684 |
| Women | 51 (40, 58) | 46 (38, 54) | 58 (50, 61) | 40 (38, 44) | 0.215 |

Footnote: * Data are given as n (%) or mean (SD). Abbreviations: LV = left ventricle, T1 = myocardial longitudinal relaxation time. Area-at-risk was measured with T2-mapping. Data are reported as mean (SD), median (IQR), or N (%) as appropriate. P-values have been obtained from a one-way ANOVA or Fisher test.

# Supplementary Discussion

We found that a hypointense infarct core revealed by native T1 mapping and microvascular obstruction revealed by late gadolinium imaging, assessed independently by different observers, co-existed in 86% of patients implying a common pathological basis. The positive predictive value of early (dynamic) microvascular obstruction for native T1 infarct core was higher (93.8%) than that of late microvascular obstruction (85.6%). This difference can be explained by the occurrence of a hypointense T1 core in close association with microvascular obstruction in the early gadolinium enhancement imaging but a hypointense T1 core is less strongly associated with microvascular obstruction in the late gadolinium enhancement imaging. High negative predictive values were observed for both late microvascular obstruction and T2* core (93.8% and 97.9%, respectively) for a native T1 core (Supplementary Table 3). Early microvascular obstruction is to some extent a dynamic pathology since it may dissipate over time due to the contribution of reversible oedema and microvascular spasm. Native T1 is also affected by these pathologies, hence its closer association with early microvascular obstruction than with late microvascular obstruction which is a more persistent pathology because of its association with irreversible capillary destruction and intra-myocardial haemorrhage [35,36]. This thesis merits further assessment in pathology studies.

Our study builds on the results from previous studies of infarct core pathology [12-16]. Dall’Armelina *et al.* studied 41 acute MI patients and found that native T1 values correlated with the segmental extent of MI and LV function acutely and with improvements in LV function at 6 months [15]. However, their study had some limitations. There were no age- or sex-matched controls, the sample size was limited (n=32 STEMI patients) so multivariable analyses were not performed, and 17% of the cohort did not have follow-up imaging. In some of their analyses, segments with microvascular obstruction were not included, Our study differed in a number of important ways from that of Dall’Armelina *et al.* [15]. First, our STEMI cohort was 10-fold larger in size, and 7% had primary reperfusion therapy with thrombolysis. We used a different T1-mapping method and CMR was performed at 1.5 Tesla rather than 3.0 Tesla (which is associated with higher T1 values) [41]. We assessed T1 values in all patients and specifically focused on patients with microvascular obstruction rather than excluding them. We also performed multivariable analyses to assess the prognostic significance of T1 values for LV outcomes, independent of clinical characteristics, including LV volume and the ischaemic area-at-risk.

The native T1 values of healthy subjects in the current study (men = 949±20 ms, women = 968±23 ms) were similar to T1 values reported by Piechnik *et al*. (men = 947±20 ms and women = 974±25 ms) [38], although lower than the study by Liu *et al.* (men = 962±37 and women = 984±47 ms) [42]; however the cohort studied by Liu *et al.* [42] had several cardiovascular risk factors and hypertension and diabetes were prevalent.
